# 1000 Genomes-based meta-analysis identifies 10 novel loci for kidney function

**DOI:** 10.1038/srep45040

**Published:** 2017-04-28

**Authors:** Mathias Gorski, Peter J. van der Most, Alexander Teumer, Audrey Y. Chu, Man Li, Vladan Mijatovic, Ilja M. Nolte, Massimiliano Cocca, Daniel Taliun, Felicia Gomez, Yong Li, Bamidele Tayo, Adrienne Tin, Mary F. Feitosa, Thor Aspelund, John Attia, Reiner Biffar, Murielle Bochud, Eric Boerwinkle, Ingrid Borecki, Erwin P. Bottinger, Ming-Huei Chen, Vincent Chouraki, Marina Ciullo, Josef Coresh, Marilyn C. Cornelis, Gary C. Curhan, Adamo Pio d’Adamo, Abbas Dehghan, Laura Dengler, Jingzhong Ding, Gudny Eiriksdottir, Karlhans Endlich, Stefan Enroth, Tõnu Esko, Oscar H. Franco, Paolo Gasparini, Christian Gieger, Giorgia Girotto, Omri Gottesman, Vilmundur Gudnason, Ulf Gyllensten, Stephen J. Hancock, Tamara B. Harris, Catherine Helmer, Simon Höllerer, Edith Hofer, Albert Hofman, Elizabeth G. Holliday, Georg Homuth, Frank B. Hu, Cornelia Huth, Nina Hutri-Kähönen, Shih-Jen Hwang, Medea Imboden, Åsa Johansson, Mika Kähönen, Wolfgang König, Holly Kramer, Bernhard K. Krämer, Ashish Kumar, Zoltan Kutalik, Jean-Charles Lambert, Lenore J. Launer, Terho Lehtimäki, Martin de Borst, Gerjan Navis, Morris Swertz, Yongmei Liu, Kurt Lohman, Ruth J. F. Loos, Yingchang Lu, Leo-Pekka Lyytikäinen, Mark A. McEvoy, Christa Meisinger, Thomas Meitinger, Andres Metspalu, Marie Metzger, Evelin Mihailov, Paul Mitchell, Matthias Nauck, Albertine J. Oldehinkel, Matthias Olden, Brenda WJH Penninx, Giorgio Pistis, Peter P. Pramstaller, Nicole Probst-Hensch, Olli T. Raitakari, Rainer Rettig, Paul M. Ridker, Fernando Rivadeneira, Antonietta Robino, Sylvia E. Rosas, Douglas Ruderfer, Daniela Ruggiero, Yasaman Saba, Cinzia Sala, Helena Schmidt, Reinhold Schmidt, Rodney J. Scott, Sanaz Sedaghat, Albert V. Smith, Rossella Sorice, Benedicte Stengel, Sylvia Stracke, Konstantin Strauch, Daniela Toniolo, Andre G. Uitterlinden, Sheila Ulivi, Jorma S. Viikari, Uwe Völker, Peter Vollenweider, Henry Völzke, Dragana Vuckovic, Melanie Waldenberger, Jie Jin Wang, Qiong Yang, Daniel I. Chasman, Gerard Tromp, Harold Snieder, Iris M. Heid, Caroline S. Fox, Anna Köttgen, Cristian Pattaro, Carsten A. Böger, Christian Fuchsberger

**Affiliations:** 1 Department of Genetic Epidemiology, University Regensburg, Regensburg, Germany; 2 Department of Nephrology, University Hospital Regensburg, Regensburg, Germany; 3 Department of Epidemiology, University of Groningen, University Medical Center Groningen, P.O. box 30.001, 9700 RB Groningen, The Netherlands; 4 Institute for Community Medicine, University Medicine Greifswald, Walther-Rathenau-Str. 48, 17475 Greifswald, Germany; 5 NHLBI’s Framingham Heart Study, Framingham, MA 01702, USA; 6 Division of Preventive Medicine, Brigham and Women’s Hospital and Harvard Medical School, Boston, MA, 02215, USA; 7 Department of Epidemiology, Johns Hopkins Bloomberg School of Public Health, 615N Wolfe St, Baltimore, MD, 21205, USA; 8 Division of Nephrology and Department of Human Genetics, University of Utah, USA; 9 Department of Life and Reproduction Sciences, University of Verona, Strada Le Grazie 8, 37134, Verona, Italy; 10 Division of Genetics and Cell Biology, San Raffaele Scientific Institute, 20132, Milano, Italy; 11 Department of Medical, Surgical and Health Sciences, University of Trieste, 34100, Trieste, Italy; 12 Center for Biomedicine, European Academy of Bozen/Bolzano (EURAC), affiliated to the University of Lübeck, Bolzano, Italy; 13 Division of Statistical Genomics, Department of Genetics, Washington University School of Medicine, St Louis, MO, 63108, USA; 14 Division of Genetic Epidemiology, Medical Center and Faculty of Medicine - University of Freiburg, Freiburg, Germany; 15 Loyola University Chicago, 2160 South First Avenue, Bldg 105, Maywood, IL 60153, USA; 16 Icelandic Heart Association, Kopavogur, Iceland; 17 University of Iceland, Reykjavik, Iceland; 18 School of Medicine and Public Health, University of Newcastle, Australia; 19 Public Health Program, Hunter Medical Research Institute, Newcastle, New South Wales, Australia; 20 Clinic for Prosthodontic Dentistry, Gerostomatology and Material Science, University Medicine Greifswald, Ferdinand-Sauerbruch-Str., 17475 Greifswald, Germany; 21 Institute of Social and Preventive Medicine, Lausanne University Hospital (CHUV), Route de la Corniche 10, 1010, Lausanne, Switzerland; 22 University of Texas Health Science Center at Houston, USA; 23 Regeneron Genetics Center, Regeneron Pharmaceuticals, Tarrytown, NY, USA; 24 The Charles Bronfman Institute for Personalized Medicine, Ichan School of Medicine at Mount Sinai, USA; 25 Inserm U1167, Lille University, Institut Pasteur de Lille, Lille, France; 26 Institute of Genetics and Biophysics, “Adriano Buzzati-Traverso”-CNR, Via P. Castellino 111, 80131 Napoli, Italy; 27 IRCCS Neuromed, via dell’Elettronica, Pozzilli (Is), Italy; 28 Department of Preventive Medicine, Northwestern University Feinberg School of Medicine, 680 N Lake Shore Drive, Suite 1400 Chicago, IL 60611, USA; 29 Renal Division, Brigham and Women’s Hospital, USA; 30 Channing Division of Network Medicine, Brigham and Women’s Hospital, Boston, MA, USA; 31 Clinical Department of Medical, Surgical and Health Science, University of Trieste, Italy; 32 Department of Epidemiology, Erasmus Medical Center, Rotterdam, The Netherlands; 33 Wake Forest School of Medicine, USA; 34 Institute of Anatomy and Cell Biology, University Medicine Greifswald, Friedrich-Loeffler-Str. 23c, 17475 Greifswald, Germany; 35 Department of Immunology, Genetics, and Pathology, Biomedical Center, SciLifeLab Uppsala, Uppsala University, SE-75108 Uppsala, Sweden; 36 Estonian Genome Center, University of Tartu, Tartu, Estonia; 37 Department of Medical Sciences, Chirurgical and Health Department, University of Trieste, Trieste, Italy; 38 Institute for Maternal and Child Health - IRCCS “Burlo Garofolo”, Trieste, Italy; 39 Institute of Genetic Epidemiology, Helmholtz Zentrum München, German Research Center for Environmental Health, Ingolstädter Landstr. 1, 85764 Neuherberg, Germany; 40 Research Unit of Molecular Epidemiology, Helmholtz Zentrum München - German Research Center for Environmental Health, Neuherberg, Germany; 41 Institute of Epidemiology II, Helmholtz Zentrum München, German Research Center for Environmental Health, Ingolstädter Landstr. 1, 85764 Neuherberg, Germany; 42 Faculty of Medicine, University of Iceland, Reykjavik, Iceland; 43 Health Services Research Group, University of Newcastle, Australia; 44 Intramural Research Program, Laboratory of Epidemiology and Population Studies, National Institute on Aging, USA; 45 INSERM, Centre INSERM Research Center U1219, Bordeaux, France; 46 University Bordeaux, ISPED, Bordeaux, France; 47 Clinical Division of Neurogeriatrics, Department of Neurology, Medical University of Graz, Austria; 48 Institute of Medical Informatics, Statistics and Documentation, Medical University of Graz, Austria; 49 Interfaculty Institute for Genetics and Functional Genomics, University Medicine Greifswald, Friedrich-Ludwig-Jahn-Str. 15a, 17475 Greifswald, Germany; 50 Department of Nutrition, Harvard School of Public Health and Channing Division of Network Medicine, Brigham and Women’s Hospital, USA; 51 German Center for Diabetes Research (DZD), Neuherberg, Germany; 52 Department of Pediatrics, Faculty of Medicine and Life Sciences, University of Tampere, Tampere 33014, Finland; 53 Unit Chronic Disease Epidemiology, Swiss Tropical and Public Health Institute, Basel, Switzerland; 54 University of Basel, Switzerland; 55 Department of Clinical Physiology, Tampere University Hospital, Tampere 33521, Finland; 56 Department of Clinical Physiology, Faculty of Medicine and Life Sciences, University of Tampere, Tampere 33014, Finland; 57 Deutsches Herzzentrum München, Technische Universität München, Munich, Germany; 58 DZHK (German Centre for Cardiovascular Research), partner site Munich Heart Alliance, Munich, Germany; 59 Department of Internal Medicine II - Cardiology, University of Ulm Medical Center, Ulm, Germany; 60 University Medical Centre Mannheim, 5th Department of Medicine, University of Heidelberg, Theodor Kutzer Ufer 1–3, 68167 Mannheim, Germany; 61 Institute of Environmental Medicine, Karolinska Institute, Stockholm, Sweden; 62 Department of Clinical Chemistry, Fimlab Laboratories, Tampere 33520, Finland; 63 Department of Clinical Chemistry, Faculty of Medicine and Life Sciences, University of Tampere, Tampere 33014, Finland; 64 University Medical Center Groningen, University of Groningen, The Netherlands; 65 The Mindich Child Health Development Institute, Icahn School of Medicine at Mount Sinai, USA; 66 Institute of Human Genetics, Helmholtz Zentrum München, German Research Center for Environmental Health, Neuherberg, Germany; 67 Institute of Human Genetics, Technische Universität München, Munich, Germany; 68 Inserm U1018, University Paris-Sud, UVSQ, University Paris-Saclay, Villejuif, France; 69 Centre for Vision Research, Department of Ophthalmology and Westmead Institute for Medical Research, University of Sydney C24, NSW, 2145, Australia; 70 Institute of Clinical Chemistry and Laboratory Medicine-University Medicine Greifswald, Ferdinand-Sauerbruch-Str., 17475 Greifswald, Germany; 71 DZHK (German Center for Cardiovascular Research), partner site Greifswald, Greifswald, Germany; 72 Department of Psychiatry, University of Groningen, University Medical Center Groningen, P.O. box 30.001, 9700 RB, Groningen, The Netherlands; 73 Department of Psychiatry, Vrije Universiteit, VU University Medical Center, NESDA, A.J. Ernststraat 1187, 1081HL Amsterdam, The Netherlands; 74 Department of Clinical Physiology and Nuclear Medicine, Turku University Hospital, Turku 20521, Finland; 75 Research Centre of Applied and Preventive Cardiovascular Medicine, University of Turku, Turku 20520, Finland; 76 Institute of Physiology, University Medicine Greifswald, 17475 Greifswald, Germany; 77 Division of Cardiovascular Medicine, Brigham and Women’s Hospital and Harvard Medical School, Boston MA 02115, USA; 78 Department of Internal Medicine, Erasmus Medical Center, Rotterdam, The Netherlands; 79 Joslin Diabetes Center. Harvard Medical School, Boston, MA, USA; 80 Institute of Molecular Biology and Biochemistry, Centre for Molecular Medicine, Medical University of Graz, Austria; 81 School of Biomedical Sciences and Pharmacy, University of Newcastle, Australia; 82 Molecular Medicine, Pathology North Ph. 0409926764, Newcastle, Australia; 83 Clinic for Internal Medicine A, University Medicine Greifswald, Ferdinand-Sauerbruch-Str., 17475 Greifswald, Germany; 84 Institute of Medical Informatics, Biometry and Epidemiology, Chair of Genetic Epidemiology, Ludwig-Maximilians-Universität, Munich, Germany; 85 Division of Medicine, Turku University Hospital, Turku 20521, Finland; 86 Department of Medicine, University of Turku, Turku 20520, Finland; 87 Department of Internal Medicine, Lausanne University Hospital (CHUV), Lausanne, Switzerland; 88 DZD (German Center for Diabetes Research), Site Greifswald, Greifswald, Germany; 89 Department of Biostatistics, Boston University School of Public Health, 715 Albany Street, Boston, MA 02118, USA; 90 Division of Genetics, Brigham and Women’s Hospital and Harvard Medical School, Boston MA, USA; 91 Broad Institute of MIT and Harvard, Cambridge MA 02142 USA; 92 Weis Center for Research, Geisinger Clinic, Danville, Pennsylvania, USA; 93 Department of Epidemiology, Johns Hopkins Bloomberg School of Public Health, Baltimore, USA

## Abstract

HapMap imputed genome-wide association studies (GWAS) have revealed >50 loci at which common variants with minor allele frequency >5% are associated with kidney function. GWAS using more complete reference sets for imputation, such as those from The 1000 Genomes project, promise to identify novel loci that have been missed by previous efforts. To investigate the value of such a more complete variant catalog, we conducted a GWAS meta-analysis of kidney function based on the estimated glomerular filtration rate (eGFR) in 110,517 European ancestry participants using 1000 Genomes imputed data. We identified 10 novel loci with p-value < 5 × 10^−8^ previously missed by HapMap-based GWAS. Six of these loci (*HOXD8*, *ARL15*, *PIK3R1*, *EYA4*, *ASTN2*, and *EPB41L3*) are tagged by common SNPs unique to the 1000 Genomes reference panel. Using pathway analysis, we identified 39 significant (FDR < 0.05) genes and 127 significantly (FDR < 0.05) enriched gene sets, which were missed by our previous analyses. Among those, the 10 identified novel genes are part of pathways of kidney development, carbohydrate metabolism, cardiac septum development and glucose metabolism. These results highlight the utility of re-imputing from denser reference panels, until whole-genome sequencing becomes feasible in large samples.

Chronic kidney disease (CKD) is a major public health concern affecting ~10% of the global adult population^1^. CKD is defined based on the glomerular filtration rate estimated from serum creatinine (eGFRcrea), a quantitative phenotype for which 53 loci have been identified so far by meta-analyses of genome-wide association studies (GWAS)^2^,^3^,^4^,^5^,^6^,^7^. These GWAS meta-analyses were based on ~2.5 million variants imputed from the HapMap Project reference panel^8^. Similar to the genetic variants identified for other phenotypes, all variants associated with eGFRcrea had a minor allele frequency (MAF) of >5%. However, though heritability of eGFR has been estimated in family studies to range between 36–75%^9^,^10^, the identified variants explain less than 4% of the variance of eGFRcrea^7^ and are located in regions of extended linkage disequilibrium (LD). So far, causal genes or variants have only been identified for a few of the association signals^11^,^12^.

It has been shown that variants poorly tagged by GWAS arrays and HapMap imputation, particularly low-frequency variants (1% ≤ MAF ≤ 5%), can explain additional variability^13^. Recent technological advances resulted in large collections of whole-genome sequence data, such as those from The 1000 Genomes project^14^,^15^. These data provide better coverage and increased imputation quality compared to previous HapMap imputation^16^, particularly for low-frequency variants.

We undertook a meta-analysis of GWAS from 33 studies that imputed genotypes from The 1000 Genomes reference panel, hypothesizing that this would uncover novel common variants associated with eGFRcrea, extend to low-frequency variants, reveal novel pathways of eGFRcrea associated genes, and improve fine-mapping of known eGFRcrea loci previously identified by our HapMap-based GWAS^3^,^4^,^5^,^6^,^7^.

## Results

### Study characteristics

In total, 110,517 adult individuals of European ancestry from 33 studies participated in GWAS meta-analysis of eGFRcrea using genotypes imputed with The 1000 Genomes Phase I reference panel^14^ (1000 Genomes meta-analysis). In addition, we performed a GWAS meta-analysis of eGFR derived from cystatin C (eGFRcys), an alternative marker of kidney function available in 11 of the 33 studies (n = 24,063). Participating studies, phenotypic characteristics, genotype information, and methods of analysis are reported in Supplementary Tables 1, 2, 3 and 4, respectively. The 1000 Genome meta-analysis results on eGFRcrea are compared with our previously published HapMap imputed data^7^, which was a HapMap-based meta-analysis of 133,814 European ancestry individuals from 50 studies.

### Imputation quality of variants imputed with The 1000 Genomes reference panel

The 1000 Genomes meta-analysis consisted of 10,971,307 genetic variants (10,159,097 SNPs and 812,210 insertion-deletions) with imputation quality IQ > 0.4^17^ in each of the studies and present in at least 50% of the subjects. Depending on the imputation methodology used, the IQ was reported as RSQ^18^ or info-score^19^ (Supplementary Table 3). Compared to the HapMap meta-analysis, the 1000 Genomes meta-analysis included a higher number of well imputed variants (8,103,124 versus 2,249,027 variants with IQ > 0.8), particularly among the low-frequency variants (1,585,176 versus 191,580, Supplementary Table 5). While rare variants (MAF ≤ 1%) were not available in the previous HapMap meta-analysis, there were even 632,526 well-imputed rare variants in the 1000 Genomes meta-analysis. When limiting the comparison to variants available in both panels, the proportion of well-imputed variants was higher in the 1000 Genomes compared to the HapMap meta-analysis (96.9% versus 93.3% for all; 88.3% versus 78.4% for the less frequent variants, Supplementary Table 5).

### 1000 Genomes meta-analysis results

**Figure 1:**
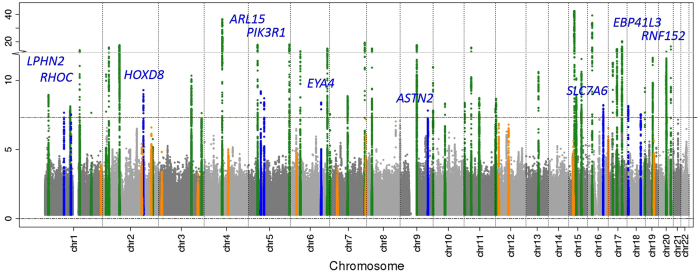
Manhattan Plot of the results of the 1000 Genome meta-analysis of eGFRcrea. Shown are the (−log10) p-values by genomic position (GRCh build 37). Highlighted are the 10 novel loci identified with genome-wide significance (blue, annotated by nearest gene), the 39 previously published^2^,^3^,^4^,^5^,^6^,^7^ and confirmed (genome-wide significant) loci (green) and the 14 previously published loci that were not genome-wide significant in this analysis (orange).

**Figure 2:**
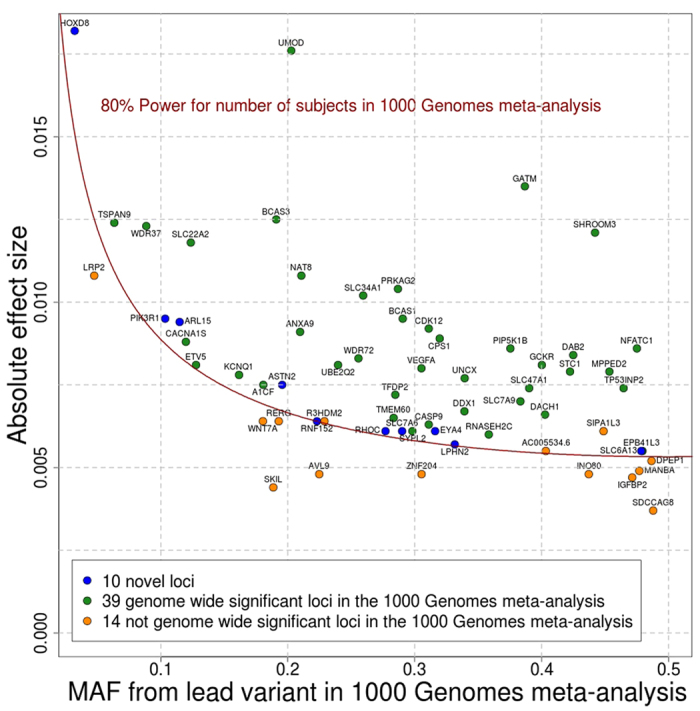
Effects of the 1000 Genomes lead variants for all novel and known loci. Shown are the effect sizes and minor allele frequencies (MAF) of the 1000 Genomes lead variants (variants with smallest p-value) in each of the 10 novel (blue), the 39 known genome-wide significant loci (green), and the 14 known loci that were not genome-wide significant in this analysis (orange). Additionally, the 80% power to detect such effects in a sample size of 110,000 subjects (as in this 1000 Genomes meta-analysis) is shown as a red line. A known locus is defined by the published lead variant ±1 Mb; a novel locus is defined by the 1000 Genome lead variant ±1 Mb.

The 1000 Genomes meta-analysis identified 49 genome-wide significant loci for eGFRcrea including 10 novel loci (lead variant p-value < 5 × 10^−8^, Table 1, Fig. 1, **and** Supplementary Figure 1). All identified lead variants were SNPs, and all were common, except rs187355703 near *HOXD8* (MAF = 0.03). None of the novel loci contained genes known to cause monogenic forms of kidney disease and for most genes no connection to kidney function or kidney disease has yet been described (Supplementary Table 6). However, it should be acknowledged that genetic variants identified in GWAS are not necessarily associated with the function of the physically closest gene. Of the 53 known eGFRcrea loci identified previously based on HapMap^2^,^3^,^4^,^5^,^6^,^7^, 39 were also genome-wide significant in the current 1000 Genomes meta-analysis (Supplementary Table 7) and the remaining 14 showed directions of association consistent with published reports, but did not reach significance (p-values 2.2 × 10^−2^ to 5.2 × 10^−7^; Supplementary Table 8). These results are consistent with our expectations from power computations (Fig. 2). Among the 39 lead variants in previously published loci that were genome-wide significant in the 1000 Genomes meta-analysis, 6 lead variants were found to be the same as the previously published variants, 25 were highly correlated (r^2^ > 0.6), and 8 showed moderate or no correlation (r^2^ ≤ 0.6).

The 1000 Genomes meta-analysis of eGFRcys confirmed previously identified loci in or near *CST3/CST9* (p-value = 4.1 × 10^−153^), *UMOD* (p-value = 2.9 × 10^−10^), and *ATXN2* (p-value = 1.6 × 10^−8^), but did not reveal any novel signal.

### The ten novel eGFRcrea loci in the context of the different reference panels

For six of the ten novel loci (*HOXD8*, *ARL15*, *PIK3R1*, *EYA4*, *ASTN2*, and *EPB41L3*), the lead variant identified in the 1000 Genomes meta-analysis was not observed in any previous HapMap meta-analysis and in fact was not genotyped as part of the HapMap reference panel. Moreover, no variant in LD with any of these six lead variants (r^2^ or D’ ≥ 0.4) was available in the HapMap panel. These loci have been missed due to the limited coverage of the HapMap panel.

For one further locus, *RHOC*, the 1000 Genome meta-analysis lead variant was present also in our previous HapMap meta-analysis, but with a lower imputation quality (1000 Genomes median IQ across all studies of 0.96 versus HapMap median IQ of 0.86). The effect size was slightly higher in the 1000 Genomes compared to the HapMap meta-analysis (0.0061 versus 0.0051 ln ml/min/1.73 m^2^, Supplementary Table 9). This locus might have been missed in the HapMap meta-analysis due to the higher uncertainty in the imputed genotypes, which is known to diminish power and to attenuate effect size in linear regression^20^.

For the remaining three loci (*LPHN2*, *SLC7A6* and *RNF152*), the lead variants of the 1000 Genomes meta-analysis were observed in the HapMap meta-analysis and similarly well imputed (IQ near 1.0 for both panels). The effect sizes were similar for all three SNPs in both 1000 Genomes and HapMap meta-analyses (0.0057 versus 0.0041, 0.0061 versus 0.0049, 0.0064 versus 0.0050 ln ml/min/1.73 m^2^ respectively) and the HapMap estimates lie well within the 98.5% confidence interval of the 1000 Genomes estimates. No substantial between-study heterogeneity was observed (I^2^ = 19%, 0%, or 21%, respectively, Supplementary Table 9). Since the p-values in the HapMap analysis were just short of genome-wide significance (p-values 8.38 × 10^−6^ to 2.33 × 10^−7^; type II error of 14–29%), it is conceivable that these variants have been missed previously by chance.

### Pathway analyses

Data-driven Expression Prioritized Integration for complex Traits (DEPICT)^21^ analysis of eGFRcrea identified 39 significant (FDR < 0.05) genes and 127 significantly (FDR < 0.05) enriched gene sets that were not identified previously^7^. Among those, 23 gene sets contained at least one of the 10 novel index genes as a top 10 hit, underpinning the influence of ureteric bud morphogenesis on kidney development and the influence of abnormal glucose homeostasis and glucan metabolic process on carbohydrate metabolism (Supplementary Table 10). All 127 significant gene sets were further grouped into meta gene sets, corresponding to their correlation of gene expression. The two most significant meta gene sets were Cardiac Septum Development (p-value = 4.48 * 10^−5^) and Glucose Metabolism (p-value = 6.11 * 10^−5^), containing one of the 10 novel index genes (Supplementary Figure 2). We repeated the analysis with varying parameters (50, 200, and 500 repetitions and 500, 2000, and 5000 permutations, respectively), confirming our primary top gene sets at an FDR of <0.05. P-values ranged from 1.32 × 10^−3^ to 4.48 × 10^−5^ and from 8.27 × 10^−4^ to 4.98 × 10^−5^ for Cardiac Septum Development and Glucose Metabolism, respectively. We replicated also the strong influence of embryonic development, kidney transmembrane transporter activity, and kidney and urogenital system morphology in the genesis of CKD from our previous findings^7^: enrichment of all 148 previously identified gene sets was nominally significant (p-value < 0.05).

### Independent association signals at novel and known loci

To identify independent association signals within a known or novel locus, we performed joint conditional analysis of eGFRcrea based on aggregated study-specific statistics using the GCTA software^22^. Among the combined 49 loci (39 known and 10 novel) attaining genome-wide significance, we uncovered eight independent signals, all among the previously reported loci, with p-values ranging from 2.39 × 10^−8^ to 2.78 × 10^−17^ after conditioning on the lead variants at each locus (Supplementary Table 11 and Supplementary Figure 3). We found that in all but one locus (*DDX1*), the previously reported lead variant was also genome-wide significant in our 1000 Genomes meta-analysis. A more detailed reasoning for the independent association signals is proposed in Supplementary Table 12. Information about biological knowledge of the highlighted genes is presented in Supplementary Table 13.

### Proportion of phenotypic variance explained and polygenic risk score (PRS) analysis

The overall proportion of phenotypic variance of eGFRcrea explained by the lead variants of the 1000 Genomes meta-analysis in all novel and known loci was 3.99%: 0.46% by the 10 lead variants in the novel loci, 3.12% by the 39 lead variants in the known loci, and 0.41% by the 1000 Genomes lead variants in the 14 known loci that were not genome-wide significant in this analysis.

Next, we tested the proportion of eGFRcrea variance that could be explained by common genetic variants in 1,071 independent adolescents participating in the TRAILS study. Given prior evidence that eGFRcrea-associated genes are preferentially expressed in the kidney and enriched for genes important in kidney development^23^, external influences on eGFRcrea such as those for the two main drivers of CKD, diabetes and hypertension, may be less important in this setting. In TRAILS, the maximum proportion of variance explained by SNPs associated at pre-defined p-value thresholds was 2.2% for a PRS composed of SNPs associated with eGFRcrea at p-value < 1 × 10^−5^ (Supplementary Table 14).

### SNP-based heritability analysis

The heritability estimate using variants of MAF > 0.01 for eGFRcrea in the ARIC study was 0.21 (95% CI 0.14–0.28) and 0.31 (95% CI 0.20–0.41) for all variants. This is in line with estimates in the literature from population-based family studies such as the Framingham Heart Study (adjusted h^2^ 0.33, 95% CI 0.19–0.47)^24^.

### Expression quantitative trait loci (eQTL) lookup

To explore potential functional implications of the novel loci, we interrogated published databases of *cis* eQTL in whole blood^25^ for the significant SNPs or their proxy variants (r^2^ > 0.8 within a 1 MB window). At 2 novel loci, significant association (p-value < 0.004) with gene expression were found: rs1111571 with *SLC7A6*, *ZFP90*, *LYPLA3* and *NFATC3*, and for rs12144044 with *RHOC* and *ST7L* (Supplementary Table 15).

We expanded our downstream analysis by annotating the significant variants with known and predicted regulatory elements using Regulome DB^26^: We confirmed rs1111571 and rs12144044 as significant associations with gene expression and found supporting evidence that these two variants show also evidence for transcription factor binding sites and DNase peaks. For the locus identified by rs187355703 no proxy was found for lookup.

### Genetic correlation

To investigate the genetic correlation of serum creatinine with related phenotypes, we queried LD Hub^27^ and identified modest genetic correlation with metabolic syndrome traits such as HDL, LDL, Type 2 diabetes, fasting glucose, BMI, and waist (LD score regression genetic correlation between −0.07 and 0.05). Little evidence for kidney damage is reported for a risk score of SNPs which are significant predictors of blood pressure^28^.

## Discussion

The main finding of our study is that imputing from denser and larger reference panels is a valid strategy to advance gene mapping even when the sample size cannot be increased. Using genotype imputation based on The 1000 Genomes panel led to the identification of 10 novel genome-wide significant loci for kidney function that were missed by earlier HapMap-imputed GWAS of larger sample size, partly due to the enhanced coverage of genomic variation. This phenomenon was observed in similar analyses of other phenotypes^29^. Still, it needs to be acknowledged that the additional proportion of trait variance explained by these new loci is moderate, which is also in line with findings from GWAS of other phenotypes^30^.

There are several methodological insights that can be gained from our analyses. First, this 1000 Genomes-based meta-analysis of 110,517 individuals has identified 10 novel loci and 8 independent association signals in known loci that were missed by our latest HapMap based analysis^7^. Our detailed dissection shows that 1000 Genomes imputation (i) provides variants missed or poorly tagged by HapMap based analysis and (ii) achieves a higher effective sample size through increased imputation quality.

Second, although the 1000 Genomes imputation enables the analysis of low-frequency variants, insertions and deletions, all identified top variants were SNPs, and all but one (near *HOXD8*) were common. Moreover, we did not identify any low-frequency variant of large effect. Our results are highly concordant with those of other recent complex diseases studies^31^ showing that low-frequency variants are also contributing to complex disease risk, but that most observed effect sizes are small or modest, and hundreds of thousands of subjects are required for detection. To identify the contribution of rare variants (MAF < 1%) to eGFRcrea, large-scale sequencing data in addition to genomic chip data have been shown to be a promising approach^31^.

Third, these novel loci, missed by our previous analysis^7^, extend our knowledge of pathways underlying kidney function, which depicts the influence of kidney development, kidney structure, and metabolic activity on the development of CKD.

The comparison of our 1000 Genomes meta-analysis with our previous HapMap meta-analysis is limited by several factors: the current analysis consists of a reduced number of samples and a slightly different study composition. Furthermore, different 1000 Genomes reference panels were used to impute genotypes and advances in imputation software and methodology must be acknowledged^32^,^33^. Nevertheless, six of the ten lead variants in the novel loci are only covered by The 1000 Genomes reference panels, which demonstrates the advantage of meta-analyses on 1000 Genomes over HapMap imputed genotypes.

In conclusion, we identified 10 novel loci and 8 additional independent association variants within known loci associated with kidney function and identified 127 novel pathways for kidney function. These results highlight the utility of re-imputing studies from improved reference panels as an intermediate cost-efficient approach to scan the full allelic frequency range for kidney function associated variants, until whole genome sequencing is feasible in large samples.

## Methods

### Phenotype definition

Each study measured serum creatinine as described in Supplementary Table 1. Between-laboratory variation has been accounted for by calibrating creatinine to the US nationally representative National Health and Nutrition Examination Study (NHANES) data in all studies^4^,^34^,^35^. GFR based on serum creatinine (eGFRcrea) was estimated using the four-variable Modification of Diet in Renal Disease (MDRD) Study Equation^36^,^37^. In a subset of studies, serum cystatin C was also obtained and eGFRcys estimated as 76.7*(serum cystatin C)^−1^ 19 (see also ref. 38). The eGFRcrea and eGFRcys values < 15 ml/min/1.73 m^2^ were set to 15, and values > 200 were set to 200 ml/min/1.73 m^2^. If not stated otherwise, our presented data and results are for eGFRcrea, which was our main analysis.

### Genotyping

Genotyping was conducted in each study as specified in Supplementary Table 3. After applying appropriate quality filters, participating studies performed genotype imputation with standard imputing procedures^32^,^33^,^39^ using any version of the 1000 Genome Phase 1 reference panels. The obtained imputed genetic variants were coded as allelic dosages. Details of study specific imputation procedure and specific reference panel are given in Supplementary Table 3.

### Genome-wide association analysis

Each study performed GWAS according to a uniform analysis plan by regressing sex- and age-adjusted residuals of the natural logarithm of eGFRcrea and eGFRcys on the allelic dosage levels. When appropriate, adjustment for study-specific features such as study site or genetic principal components was included in the model. Family-based studies accounted for relatedness using mixed effect models. Details on the study-specific methods are reported in Supplementary Table 4.

### GWAS meta-analysis

All GWAS files underwent quality control using the GWAtoolbox package^40^. GWAS meta-analyses for eGFRcrea and eGFRcys were performed using the software METAL^41^ assuming fixed effects across studies and using inverse-variance weighting, excluding variants with imputation quality IQ ≤ 0.4 or variants present in less than 50% of the 110,517 subjects (yielding 10,971,307 variants). The genomic inflation factor λ was estimated for each study as the ratio between the median of all observed test statistics (b/SE)^2^ and the expected median of a chi-squared with 1 degree of freedom, with b and SE representing the effect of each SNP on ln eGFRcrea or ln eGFRcys and its standard error, respectively. Genomic-control (GC) correction^42^ was applied to p-values and SEs in case of λ > 1 (1st GC correction). To limit the possibility of false positives, a second GC correction on the aggregated results was applied after the meta-analysis. Between-study heterogeneity was assessed with the I^2^ statistic^43^.

### Definition of known and novel loci

Known loci were defined by a previously published lead variant that had shown genome-wide significant association with eGFRcrea (p-value < 5 × 10^−8^) and the genetic segment around it (lead SNP ± 1 Mb)^2^,^3^,^4^,^5^,^6^,^7^. Variants outside such segments and associated with eGFRcrea at a p-value < 5 × 10^−8^ in the 1000 Genomes meta-analysis defined the novel loci. Each novel locus was pinpointed by the lead variant with the smallest p-value ± 1 Mb.

### Comparison of 1000 Genomes and HapMap results

For the variants available in both the 1000 Genomes and HapMap meta-analyses, we compared lead variants, effect sizes, imputation quality as well as the power that we had in the data to detect the respective effects. For this comparison, we also utilized the association results of our previous HapMap meta-analysis^7^ in 50 studies including a maximum of 133,814 subjects. Power was calculated in R (www.r-project.org) for the approximate maximum number of subjects in the 1000 Genomes meta-analyses (n = 110,000) to identify the lead variants with an alpha of 5 × 10^−8^. Further, effective power, which takes into account the imputation quality of the variant, was calculated based on the effective number of subjects, which is the number of subjects per variant multiplied by the median of the imputation quality across studies.

### Pathway Analyses

Pathway analyses, comprised of pathway/gene set enrichment and tissue/cell type analyses, were performed by applying a software package called Data-Driven Expression Prioritized Integration for Complex Traits (DEPICT)^21^. DEPICT performs gene set enrichment analyses by testing whether genes in GWAS-associated loci are enriched for reconstituted versions of known molecular pathways (jointly referred to as reconstituted gene sets). The reconstitution is accomplished by identifying genes that are co-regulated with other genes in a given gene set based on a panel of 77,840 gene expression microarrays^44^. Genes that are found to be transcriptionally co-regulated with genes from the original gene set are added to the gene set, which results in the reconstitution. Several types of gene sets were reconstituted in DEPICT: 5,984 protein molecular pathways derived from 169,810 high-confidence experimentally derived protein-protein interactions^45^, 2,473 phenotypic gene sets derived from 211,882 gene-phenotype pairs from the Mouse Genetics Initiative^46^, 737 Reactome database pathways^47^, 184 Kyoto Encyclopedia of Genes and Genomes (KEGG) database pathways^48^ and 5,083 Gene Ontology database terms^49^. In total, 14,461 gene sets were assessed for enrichment in genes in associated regions. DEPICT also facilitates tissue and cell type enrichment analyses by testing whether the genes in associated regions are highly expressed in any of the 209 MeSH annotations for 37,427 microarrays on the Affymetrix U133 Plus 2.0 Array platform.

In our analysis, we used DEPICT version 1 rel194 and to be comparable to our previous analysis, included all variants reaching eGFRcrea association p-values < 1 × 10^−5^ from HapMap and 1000 Genomes imputed data with genomic coordinates defined by genome build GRCh38 (https://genome.ucsc.edu/cgi-bin/hgLiftOver). Since 1000 Genomes imputed loci in the DEPICT analysis differed slightly from the HapMap imputed loci, our HapMap and 1000 Genomes input was created by adding all significant 1000 Genomes variants to all significant HapMap variants. This process resulted in a total of 3,659 variants for HapMap, 7,894 variants for 1000 Genomes, and 9,270 variants for HapMap and 1000 Genomes analyses. Next, independent lead variants were identified with Plink^50^ using ± 500 kb flanking regions and r^2^ > 0.01 with the 1000 Genomes data^14^ as reference. Genomic intervals are generated consisting of all variants within r^2^ > 0.5 to each lead variant. If any of the 19,987 genes in the analysis overlaps or resides within a genomic interval, it is mapped to that interval. After merging of overlapping regions and excluding regions within the major histocompatibility complex on chromosome 6, base pairs 25,000,000–35,000,000, DEPICT analyses were conducted using the following parameters: 200 repetitions to compute FDR and 2,000 permutations to compute p-values adjusted for gene length by using 500 null GWAS. For the enrichment analysis we used 10,968 reconstituted gene sets. For visualization, all novel significant gene sets were further merged into meta gene sets by running an affinity propagation^51^ from Pythons scikit-learn package (http://scikit-learn.org/). The network was visualized with Cytoscape (http://cytoscape.org/).

### Identification of independent association signals with GCTA

We searched for independent association signals in the known and novel loci with a joint conditional analysis on the aggregated meta-analysis results using the GCTA-COJO method (conditional and joint genome-wide association analysis)^22^,^52^. The KORA-F4 GWAS data^53^ were used to estimate the LD (r^2^) in the joint conditional analysis, and to quantify the extent of coinheritance (D’)^50^. A potential independent association signal within a given locus was reported if the variant with the smallest conditional p-value was genome-wide significant (p-value < 5 × 10^−8^) after conditioning on the previously reported variant in a locus.

### SNP-based heritability analysis

The heritability of eGFRcrea was estimated using GCTA GREML-LDMS methods^54^ (version 1.25) with imputed genotype accounting for linkage disequilibrium. The imputed genotype was based on dosage (probability > 0.9) imputed using the 1000 Genomes Phase I reference panel and filtered by the following criteria: HWE < 1 × 10^−6^, individual missingness >5%, SNP missingness >5%, and MAF < 0.0005 (~3 copies).

### Proportion of phenotypic variance explained

To quantify the impact of the identified genetic loci on renal function, the percent of phenotypic variance explained by all lead variants in the novel and known loci was estimated as 
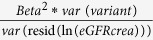
, where *var* (*variant*) = 2 * *MAF* * (1−*MAF*)and beta is the estimated effect of the variant in the 1000 Genomes meta-analysis^55^. The variance of the residuals of ln (eGFRcrea) is computed in the ARIC study (n = 9,038). All variants were assumed to have independent effects on the phenotype.

### Polygenic risk score analysis

PriorityPruner (http://prioritypruner.sourceforge.net) was used to select independent SNPs from The 1000 Genomes reference panel using an algorithm that preferentially selects SNPs that are more significant in the current 1000 Genomes meta-analysis compared to the previous HapMap meta-analysis. Polygenic risk scores (PRSs), using various thresholds of significance, as obtained from the 1000 Genomes meta-analysis results and weighted for the effects sizes within study were generated in TRAILS^56^ (n = 1,071), an independent study of adolescents, which was not part of the meta-analysis. These PRSs were tested for association with eGFRcrea using linear regression in R and the variance explained by the PRSs was calculated.

## Additional Information

**How to cite this article:** Gorski, M. *et al*. 1000 Genomes-based meta-analysis identifies 10 novel loci for kidney function. *Sci. Rep.* **7**, 45040; doi: 10.1038/srep45040 (2017).

**Publisher's note:** Springer Nature remains neutral with regard to jurisdictional claims in published maps and institutional affiliations.

## Supplementary Material

Supplementary Information

## Figures and Tables

**Table 1:** The 10 novel genome-wide significant loci (p < 5 × 10^−8^) associated with eGFRcrea in up to 110,517 subjects from up to 33 studies.

Variant ID	Chr	Position	Index Gene	Effect allele/non-effect allele	Effect allele frequency	Effect (SE)	p-value	I^2^ (%)	IQ	Number of subjects in analysis
rs10874312	1	82,944,571	*LPHN2*	A/G	0.67	−0.0057 (0.0011)	2.20 × 10^−08^	19	1.00	107,335
rs12144044	1	113,248,791	*RHOC*	A/C	0.28	−0.0061 (0.0011)	2.87 × 10^−08^	0	0.96	110,517
rs187355703	2	176,993,583	*HOXD8*	C/G	0.97	0.0182 (0.0030)	5.15 × 10^−10^	5	0.89	109,257
rs111366116	5	53,295,546	*ARL15*	T/C	0.11	0.0094 (0.0015)	6.27 × 10^−10^	22	0.97	110,517
rs113246091	5	67,739,274	*PIK3R1*	A/G	0.10	−0.0095 (0.0016)	1.98 × 10^−09^	43	0.98	110,105
rs7764488	6	133,812,872	*EYA4*	A/G	0.32	0.0061 (0.0011)	4.08 × 10^−09^	1	0.98	110,516
rs13298297	9	119,264,108	*ASTN2*	A/G	0.20	−0.0075 (0.0014)	1.53 × 10^−08^	0	0.81	110,514
rs1111571	16	68,363,181	*SLC7A6*	A/G	0.71	0.0061 (0.0011)	6.20 × 10^−09^	0	1.00	109,275
rs9962915	18	5,593,171	*EPB41L3*	T/C	0.48	−0.0055 (0.0010)	7.19 × 10^−09^	0	0.98	110,516
rs12458009	18	59,350,507	*RNF152*	T/G	0.78	−0.0064 (0.0012)	2.90 × 10^−08^	21	1.00	107,325

Positions are given on GRCh build 37. The gene closest to the variant is listed (index gene). Effect sizes are given on the log scale. IQ = Imputation quality metric computed as median of info score (ImputeV2) or RSQ (minimac) across studies. SE = standard error. I^2^ = between-study heterogeneity statistic.

## References

[b1] Eckardt K. U. . Evolving importance of kidney disease: from subspecialty to global health burden. Lancet 382, 158–69 (2013). 23727165 10.1016/S0140-6736(13)60439-0

[b2] Chambers J. C. . Genetic loci influencing kidney function and chronic kidney disease. Nat Genet 42, 373–5 (2010). 20383145 10.1038/ng.566 PMC3748585

[b3] Chasman D. I. . Integration of genome-wide association studies with biological knowledge identifies six novel genes related to kidney function. Hum Mol Genet 21, 5329–43 (2012). 22962313 10.1093/hmg/dds369 PMC3607468

[b4] Kottgen A. . Multiple loci associated with indices of renal function and chronic kidney disease. Nat Genet 41, 712–7 (2009). 19430482 10.1038/ng.377 PMC3039280

[b5] Kottgen A. . New loci associated with kidney function and chronic kidney disease. Nat Genet 42, 376–84 (2010). 20383146 10.1038/ng.568 PMC2997674

[b6] Pattaro C. . Genome-wide association and functional follow-up reveals new loci for kidney function. PLoS Genet 8, e1002584 (2012). 22479191 10.1371/journal.pgen.1002584 PMC3315455

[b7] Pattaro C. . Genetic associations at 53 loci highlight cell types and biological pathways relevant for kidney function. Nat Commun 7, 10023 (2016). 26831199 10.1038/ncomms10023 PMC4735748

[b8] International HapMap C. . Integrating common and rare genetic variation in diverse human populations. Nature 467, 52–8 (2010). 20811451 10.1038/nature09298 PMC3173859

[b9] Boger C. A. & Heid I. M. Chronic kidney disease: novel insights from genome-wide association studies. Kidney Blood Press Res 34, 225–34 (2011). 21691125 10.1159/000326901

[b10] Pattaro C. . Genome-wide linkage analysis of serum creatinine in three isolated European populations. Kidney Int 76, 297–306 (2009). 19387472 10.1038/ki.2009.135

[b11] Trudu M. . Common noncoding UMOD gene variants induce salt-sensitive hypertension and kidney damage by increasing uromodulin expression. Nat Med 19, 1655–60 (2013). 24185693 10.1038/nm.3384 PMC3856354

[b12] Yeo N. C. . Shroom3 contributes to the maintenance of the glomerular filtration barrier integrity. Genome Res 25, 57–65 (2015). 25273069 10.1101/gr.182881.114 PMC4317173

[b13] Sveinbjornsson G. . Rare mutations associating with serum creatinine and chronic kidney disease. Hum Mol Genet 23, 6935–43 (2014). 25082825 10.1093/hmg/ddu399

[b14] Genomes Project C. . An integrated map of genetic variation from 1,092 human genomes. Nature 491, 56–65 (2012). 23128226 10.1038/nature11632 PMC3498066

[b15] Genomes Project C. . A global reference for human genetic variation. Nature 526, 68–74 (2015). 26432245 10.1038/nature15393 PMC4750478

[b16] Wood A. R. . Imputation of variants from the 1000 Genomes Project modestly improves known associations and can identify low-frequency variant-phenotype associations undetected by HapMap based imputation. PLoS One 8, e64343 (2013). 23696881 10.1371/journal.pone.0064343 PMC3655956

[b17] Nikpay M. . A comprehensive 1,000 Genomes-based genome-wide association meta-analysis of coronary artery disease. Nat Genet 47, 1121–30 (2015). 26343387 10.1038/ng.3396 PMC4589895

[b18] Li Y., Willer C. J., Ding J., Scheet P. & Abecasis G. R. MaCH: using sequence and genotype data to estimate haplotypes and unobserved genotypes. Genet Epidemiol 34, 816–34 (2010). 21058334 10.1002/gepi.20533 PMC3175618

[b19] Marchini J., Howie B., Myers S., McVean G. & Donnelly P. A new multipoint method for genome-wide association studies by imputation of genotypes. Nat Genet 39, 906–13 (2007). 17572673 10.1038/ng2088

[b20] Carroll R. J. Measurement error in nonlinear models: a modern perspective. xxviii, 455 p. (Chapman & Hall/CRC, Boca Raton, FL, 2006).

[b21] Pers T. H. . Biological interpretation of genome-wide association studies using predicted gene functions. Nat Commun 6, 5890 (2015). 25597830 10.1038/ncomms6890 PMC4420238

[b22] Yang J. . Conditional and joint multiple-SNP analysis of GWAS summary statistics identifies additional variants influencing complex traits. Nat Genet 44, 369–75, S1–3 (2012). 22426310 10.1038/ng.2213 PMC3593158

[b23] Pattaro C. . Genetic associations at 53 loci highlight cell types and biological pathways relevant for kidney function. Nat Commun 7, 10023 (2016). 26831199 10.1038/ncomms10023 PMC4735748

[b24] Fox C. S. . Genomewide linkage analysis to serum creatinine, GFR, and creatinine clearance in a community-based population: the Framingham Heart Study. J Am Soc Nephrol 15, 2457–61 (2004). 15339995 10.1097/01.ASN.0000135972.13396.6F

[b25] Westra H. J. . Systematic identification of trans eQTLs as putative drivers of known disease associations. Nat Genet 45, 1238–43 (2013). 24013639 10.1038/ng.2756 PMC3991562

[b26] Boyle A. P. . Annotation of functional variation in personal genomes using RegulomeDB. Genome Res 22, 1790–7 (2012). 22955989 10.1101/gr.137323.112 PMC3431494

[b27] Zheng J. . LD Hub: a centralized database and web interface to perform LD score regression that maximizes the potential of summary level GWAS data for SNP heritability and genetic correlation analysis. Bioinformatics 33, 272–279 (2017). 27663502 10.1093/bioinformatics/btw613 PMC5542030

[b28] Ehret G. B. . The genetics of blood pressure regulation and its target organs from association studies in 342,415 individuals. Nat Genet 48, 1171–84 (2016). 27618452 10.1038/ng.3667 PMC5042863

[b29] Horikoshi M. . Discovery and Fine-Mapping of Glycaemic and Obesity-Related Trait Loci Using High-Density Imputation. PLoS Genet 11, e1005230 (2015). 26132169 10.1371/journal.pgen.1005230 PMC4488845

[b30] Visscher P. M., Brown M. A., McCarthy M. I. & Yang J. Five years of GWAS discovery. Am J Hum Genet 90, 7–24 (2012). 22243964 10.1016/j.ajhg.2011.11.029 PMC3257326

[b31] Fritsche L. G. . A large genome-wide association study of age-related macular degeneration highlights contributions of rare and common variants. Nat Genet 48, 134–43 (2016). 26691988 10.1038/ng.3448 PMC4745342

[b32] Howie B., Fuchsberger C., Stephens M., Marchini J. & Abecasis G. R. Fast and accurate genotype imputation in genome-wide association studies through pre-phasing. Nat Genet 44, 955–9 (2012). 22820512 10.1038/ng.2354 PMC3696580

[b33] Fuchsberger C., Abecasis G. R. & Hinds D. A. minimac2: faster genotype imputation. Bioinformatics 31, 782–4 (2015). 25338720 10.1093/bioinformatics/btu704 PMC4341061

[b34] Coresh J. . Calibration and random variation of the serum creatinine assay as critical elements of using equations to estimate glomerular filtration rate. Am J Kidney Dis 39, 920–9 (2002). 11979335 10.1053/ajkd.2002.32765

[b35] Fox C. S. . Predictors of new-onset kidney disease in a community-based population. JAMA 291, 844–50 (2004). 14970063 10.1001/jama.291.7.844

[b36] Levey A. S. . A more accurate method to estimate glomerular filtration rate from serum creatinine: a new prediction equation. Modification of Diet in Renal Disease Study Group. Ann Intern Med 130, 461–70 (1999). 10075613 10.7326/0003-4819-130-6-199903160-00002

[b37] Levey A. S. . Using standardized serum creatinine values in the modification of diet in renal disease study equation for estimating glomerular filtration rate. Ann Intern Med 145, 247–54 (2006). 16908915 10.7326/0003-4819-145-4-200608150-00004

[b38] Stevens L. A. . Estimating GFR using serum cystatin C alone and in combination with serum creatinine: a pooled analysis of 3,418 individuals with CKD. Am J Kidney Dis 51, 395–406 (2008). 18295055 10.1053/j.ajkd.2007.11.018 PMC2390827

[b39] Porcu E., Sanna S., Fuchsberger C. & Fritsche L. G. Genotype imputation in genome-wide association studies. Curr Protoc Hum Genet Chapter 1, Unit 1 25 (2013). 23853078 10.1002/0471142905.hg0125s78

[b40] Fuchsberger C., Taliun D., Pramstaller P. P., Pattaro C. & consortium, C. K. GWAtoolbox: an R package for fast quality control and handling of genome-wide association studies meta-analysis data. Bioinformatics 28, 444–5 (2012). 22155946 10.1093/bioinformatics/btr679

[b41] Willer C. J., Li Y. & Abecasis G. R. METAL: fast and efficient meta-analysis of genomewide association scans. Bioinformatics 26, 2190–1 (2010). 20616382 10.1093/bioinformatics/btq340 PMC2922887

[b42] Devlin B. & Roeder K. Genomic control for association studies. Biometrics 55, 997–1004 (1999). 11315092 10.1111/j.0006-341x.1999.00997.x

[b43] Higgins J. P., Thompson S. G., Deeks J. J. & Altman D. G. Measuring inconsistency in meta-analyses. BMJ 327, 557–60 (2003). 12958120 10.1136/bmj.327.7414.557 PMC192859

[b44] Fehrmann R. S. . Gene expression analysis identifies global gene dosage sensitivity in cancer. Nat Genet 47, 115–25 (2015). 25581432 10.1038/ng.3173

[b45] Lage K. . A human phenome-interactome network of protein complexes implicated in genetic disorders. Nat Biotechnol 25, 309–16 (2007). 17344885 10.1038/nbt1295

[b46] Blake J. A. . The Mouse Genome Database: integration of and access to knowledge about the laboratory mouse. Nucleic Acids Res 42, D810–7 (2014). 24285300 10.1093/nar/gkt1225 PMC3964950

[b47] Croft D. . Reactome: a database of reactions, pathways and biological processes. Nucleic Acids Res 39, D691–7 (2011). 21067998 10.1093/nar/gkq1018 PMC3013646

[b48] Kanehisa M., Goto S., Sato Y., Furumichi M. & Tanabe M. KEGG for integration and interpretation of large-scale molecular data sets. Nucleic Acids Res 40, D109–14 (2012). 22080510 10.1093/nar/gkr988 PMC3245020

[b49] Ashburner M. . Gene ontology: tool for the unification of biology. The Gene Ontology Consortium. Nat Genet 25, 25–9 (2000). 10802651 10.1038/75556 PMC3037419

[b50] Purcell S. . PLINK: a tool set for whole-genome association and population-based linkage analyses. Am J Hum Genet 81, 559–75 (2007). 17701901 10.1086/519795 PMC1950838

[b51] Frey B. J. & Dueck D. Clustering by passing messages between data points. Science 315, 972–6 (2007). 17218491 10.1126/science.1136800

[b52] Wright A. K. & Thompson M. R. Hydrodynamic structure of bovine serum albumin determined by transient electric birefringence. Biophys J 15, 137–41 (1975). 1167468 10.1016/s0006-3495(75)85797-3 PMC1334600

[b53] Wichmann H. E., Gieger C., Illig T. & Group M. K. S. KORA-gen–resource for population genetics, controls and a broad spectrum of disease phenotypes. Gesundheitswesen 67 Suppl 1, S26–30 (2005). 16032514 10.1055/s-2005-858226

[b54] Yang J. . Genetic variance estimation with imputed variants finds negligible missing heritability for human height and body mass index. Nat Genet 47, 1114–20 (2015). 26323059 10.1038/ng.3390 PMC4589513

[b55] Rosner B. Fundamentals of biostatistics. xvii, 859 p. (Brooks/Cole, Cengage Learning, Boston, 2011).

[b56] Huisman M. . Cohort profile: the Dutch ‘TRacking Adolescents’ Individual Lives’ Survey’; TRAILS. Int J Epidemiol 37, 1227–35 (2008). 18263649 10.1093/ije/dym273

