# Corrigendum: 1000 Genomes-based meta-analysis identifies 10 novel loci for kidney function

**DOI:** 10.1038/srep46835

**Published:** 2017-05-26

**Authors:** Mathias Gorski, Peter J. van der Most, Alexander Teumer, Audrey Y. Chu, Man Li, Vladan Mijatovic, Ilja M. Nolte, Massimiliano Cocca, Daniel Taliun, Felicia Gomez, Yong Li, Bamidele Tayo, Adrienne Tin, Mary F. Feitosa, Thor Aspelund, John Attia, Reiner Biffar, Murielle Bochud, Eric Boerwinkle, Ingrid Borecki, Erwin P. Bottinger, Ming-Huei Chen, Vincent Chouraki, Marina Ciullo, Josef Coresh, Marilyn C. Cornelis, Gary C. Curhan, Adamo Pio d’ Adamo, Abbas Dehghan, Laura Dengler, Jingzhong Ding, Gudny Eiriksdottir, Karlhans Endlich, Stefan Enroth, Tõnu Esko, Oscar H. Franco, Paolo Gasparini, Christian Gieger, Giorgia Girotto, Omri Gottesman, Vilmundur Gudnason, Ulf Gyllensten, Stephen J. Hancock, Tamara B. Harris, Catherine Helmer, Simon Höllerer, Edith Hofer, Albert Hofman, Elizabeth G. Holliday, Georg Homuth, Frank B. Hu, Cornelia Huth, Nina Hutri-Kähönen, Shih-Jen Hwang, Medea Imboden, Åsa Johansson, Mika Kähönen, Wolfgang König, Holly Kramer, Bernhard K. Krämer, Ashish Kumar, Zoltan Kutalik, Jean-Charles Lambert, Lenore J. Launer, Terho Lehtimäki, Martin H. de Borst, Gerjan Navis, Morris Swertz, Yongmei Liu, Kurt Lohman, Ruth J. F. Loos, Yingchang Lu, Leo-Pekka Lyytikäinen, Mark A. McEvoy, Christa Meisinger, Thomas Meitinger, Andres Metspalu, Marie Metzger, Evelin Mihailov, Paul Mitchell, Matthias Nauck, Albertine J. Oldehinkel, Matthias Olden, Brenda WJH Penninx, Giorgio Pistis, Peter P. Pramstaller, Nicole Probst-Hensch, Olli T. Raitakari, Rainer Rettig, Paul M. Ridker, Fernando Rivadeneira, Antonietta Robino, Sylvia E. Rosas, Douglas Ruderfer, Daniela Ruggiero, Yasaman Saba, Cinzia Sala, Helena Schmidt, Reinhold Schmidt, Rodney J. Scott, Sanaz Sedaghat, Albert V. Smith, Rossella Sorice, Benedicte Stengel, Sylvia Stracke, Konstantin Strauch, Daniela Toniolo, Andre G. Uitterlinden, Sheila Ulivi, Jorma S. Viikari, Uwe Völker, Peter Vollenweider, Henry Völzke, Dragana Vuckovic, Melanie Waldenberger, Jie Jin Wang, Qiong Yang, Daniel I. Chasman, Gerard Tromp, Harold Snieder, Iris M. Heid, Caroline S. Fox, Anna Köttgen, Cristian Pattaro, Carsten A. Böger, Christian Fuchsberger

Scientific Reports 7: Article number: 4504010.1038/srep45040; published online: 04 28 2017; updated: 05 26 2017

The original version of this Article contained a typographical error in the spelling of the author Martin H. de Borst, which was incorrectly given as Martin de Borst. This has now been corrected in both the PDF and HTML versions of the Article.

