# Supplementary material for: 1000 Genomes-based meta-analysis identifies 10 novel loci for kidney function
Source: Sci Rep. 2017 Apr 28;7:45040. doi: 10.1038/srep45040 (PMC5408227; doi:10.1038/srep45040)
Supplement: Supplementary Information [file srep45040-s1.pdf]

1000 Genomes-based meta-analysis identifies 10 novel loci for kidney function

Supplementary Material

Mathias Gorski[1,2,*], Peter van der Most[3,*], Alexander Teumer[4,*], Audrey Chu[5,6,*], Mandy Li[7,8], Vladan Mijatovic[9], Inga Nolte[3], Massimiliano Cocca[10,11], Daniel Taliun[12], Felicia Gomez[13], Yong Li[14], Bamidele Tayo[15], Adrienne Tin[7], Mary F Feitosa[13], Thor Aspelund[16,17], John Attia[18,19], Reiner Biffar[20], Murielle Bochud[21], Eric Boerwinkle[22], Ingrid Borecki[23], Erwin P Bottinger[24], Ming-Huei Chen[5], Vincent Chouraki[25], Marina Ciullo[26,27], Josef Coresh[7], Marilyn C Cornelis[28], Gary C Curhan[29,30], Adamo P d'Adamo[31], Abbas Dehghan[32], Laura Dengler[2], Jingzhong Ding[33], Gudny Eiriksdottir[16], Karlhans Endlich[34], Stefan Enroth[35], Tõnu Esko[36], Oscar H Franco[32], Paolo Gasparini[37,38], Christian Gieger[39,40,41], Giorgia Girotto[37,38], Omri Gottesman[24], Vilmundur Gudnason[16,42], Ulf Gyllensten[35], Stephen J Hancock[18,43], Tamara B Harris[44], Catherine Helmer[45,46], Simon Höllerer[1], Edith Hofer[47,48], Albert Hofman[32], Elizabeth G Holliday[19], Georg Homuth[49], Frank B Hu[50], Cornelia Huth[41,51], Nina Hutri-Kähönen[52], Shih-Jen Hwang[5], Medea Imboden[53,54], Åsa Johansson[35], Mika Kähönen[55,56], Wolfgang König[57,58,59], Holly Kramer[15], Bernhard K Krämer[60], Ashish Kumar[53,54,61], Zoltan Kutalik[21], Jean-Charles Lambert[25], Lenore J Launer[44], Terho Lehtimäki[62,63], Martin de Borst[64], Gerjan Navis[64], Morris Swertz[64], Yongmei Liu[33], Kurt Lohman[33], Ruth JF Loos[24,65], Yingchang Lu[24], Leo-Pekka Lyytikäinen[62,63], Mark A McEvoy[18], Christa Meisinger[41], Thomas Meitinger[66,67], Andres Metspalu[36], Marie Metzger[68], Evelin Mihailov[36], Paul Mitchell[69], Matthias Nauck[70,71], Albertine J Oldehinkel[72], Matthias Olden[1,5], Brenda W Penninx[73], Giorgio Pistis[10], Peter P Pramstaller[12], Nicole Probst-Hensch[53,54], Olli T Raitakari[74,75], Rainer Rettig[76], Paul M Ridker[6,77], Fernando Rivadeneira[78], Antonietta Robino[38], Sylvia E Rosas[79], Douglas Ruderfer[24], Daniela Ruggiero[26], Yasaman Saba[80], Cinzia Sala[10], Helena Schmidt[80], Reinhold Schmidt[47], Rodney J Scott[81,82], Sanaz Sedaghat[32], Albert V Smith[16,42], Rossella Sorice[26,27], Benedicte Stengel[68], Sylvia Stracke[83], Konstantin Strauch[39,84], Daniela Toniolo[10], Andre G Uitterlinden[78], Sheila Ulivi[38], Jorma S Viikari[85,86], Uwe Völker[49,71], Peter Vollenweider[87], Henry Völzke[4,71,88], Dragana Vuckovic[37,38], Melanie Waldenberger[40,41], Jie J Wang[69], Qiong Yang[89], Dan Chasman[6,90,91], Gerard Tromp[92], Harold Snieder[3], Iris Heid[1], Caroline Fox[5], Anna Köttgen[14,93,@], Cristian Pattaro[12,@], Carsten Böger[2,@] and Christian Fuchsberger[12,@]

*indicates joint contribution
@indicates joint oversight

1. Department of Genetic Epidemiology, University Regensburg, Regensburg, Germany.
2. Department of Nephrology, University Hospital Regensburg, Regensburg, Germany.
3. Department of Epidemiology, University of Groningen, University Medical Center Groningen, P.O. box 30.001, 9700 RB Groningen, The Netherlands.
4. Institute for Community Medicine, University Medicine Greifswald, Walther-Rathenau-Str. 48, 17475 Greifswald, Germany.
5. NHLBI's Framingham Heart Study, Framingham MA 01702, USA.
6. Division of Preventive Medicine, Brigham and Women's Hospital and Harvard Medical School, Boston MA 02215, USA.
7. Department of Epidemiology, Johns Hopkins Bloomberg School of Public Health, 615 N Wolfe St, Baltimore, MD 21205, USA.
8. Division of Nephrology and Department of Human Genetics, University of Utah, USA.
9. Department of Life and Reproduction Sciences, University of Verona, Strada Le Grazie 8, 37134 Verona, Italy.
10. Division of Genetics and Cell Biology, San Raffaele Scientific Institute, 20132 Milano, Italy.

11. Department of Medical, Surgical and Health Sciences, University of Trieste, 34100 Trieste, Italy.
12. Center for Biomedicine, European Academy of Bozen/Bolzano (EURAC)-affiliated to the University of Lübeck, Bolzano, Italy.
13. Division of Statistical Genomics, Department of Genetics, Washington University School of Medicine, St Louis, MO USA 63108.
14. Division of Genetic Epidemiology, Medical Center and Faculty of Medicine - University of Freiburg, Freiburg, Germany.
15. Loyola University Chicago, 2160 South First Avenue, Bldg 105, Maywood, IL 60153, USA.
16. Icelandic Heart Association, Kopavogur, Iceland.
17. University of Iceland, Reykjavik, Iceland.
18. School of Medicine and Public Health, University of Newcastle, Australia.
19. Public Health Program, Hunter Medical Research Institute, Newcastle, New South Wales, Australia.
20. Clinic for Prosthodontic Dentistry, Gerostomatology and Material Science, University Medicine Greifswald, Ferdinand-Sauerbruch-Str., 17475 Greifswald, Germany.
21. Institute of Social and Preventive Medicine, Lausanne University Hospital (CHUV), Route de la Corniche 10, 1010 Lausanne, Switzerland.
22. University of Texas Health Science Center at Houston, USA.
23. Analytical Genetics Group, Regeneron Genetics Center, Regeneron Pharmaceuticals, Inc.
24. The Charles Bronfman Institute for Personalized Medicine, Ichan School of Medicine at Mount Sinai, USA.
25. Inserm U1167, Lille University, Institut Pasteur de Lille, Lille, France.
26. Institute of Genetics and Biophysics, "Adriano Buzzati-Traverso"-CNR, Via P. Castellino 111, 80131 Napoli, Italy.
27. IRCCS Neuromed, Pozzilli, Isernia, Italy.
28. Department of Preventive Medicine, Northwestern University Feinberg School of Medicine, 680 N Lake Shore Drive, Suite 1400 Chicago, IL 60611, USA.
29. Renal Division, Brigham and Women's Hospital, USA.
30. Channing Division of Network Medicine, Brigham and Women's Hospital, Boston, MA, USA.
31. Clinical Department of Medical, Surgical and Health Science, University of Trieste, Italy.
32. Department of Epidemiology, Erasmus Medical Center, Rotterdam, The Netherlands.
33. Wake Forest School of Medicine, USA.
34. Institute of Anatomy and Cell Biology, University Medicine Greifswald, Friedrich-Loeffler-Str. 23c, 17475 Greifswald, Germany.
35. Department of Immunology, Genetics, and Pathology, Biomedical Center, SciLifeLab Uppsala, Uppsala University, SE-75108 Uppsala, Sweden.
36. Estonian Genome Center, University of Tartu, Tartu, Estonia.
37. Department of Medical Sciences, Chirurgical and Health Department, University of Trieste, Trieste, Italy.
38. Institute for Maternal and Child Health - IRCCS “Burlo Garofolo”, Trieste, Italy.
39. Institute of Genetic Epidemiology, Helmholtz Zentrum München, German Research Center for Environmental Health, Ingolstädter Landstr. 1, 85764 Neuherberg, Germany.
40. Research Unit of Molecular Epidemiology, Helmholtz Zentrum München - German Research Center for Environmental Health, Neuherberg, Germany.
41. Institute of Epidemiology II, Helmholtz Zentrum München, German Research Center for Environmental Health, Ingolstädter Landstr. 1, 85764 Neuherberg, Germany.

42. Faculty of Medicine, University of Iceland, Reykjavik, Iceland.
43. Health Services Research Group, University of Newcastle, Australia.
44. Intramural Research Program, Laboratory of Epidemiology and Population Studies, National Institute on Aging, USA.
45. INSERM, Centre INSERM Research Center U1219, Bordeaux, France.
46. University Bordeaux, ISPED, Bordeaux, France.
47. Clinical Division of Neurogeriatrics, Department of Neurology, Medical University of Graz, Austria.
48. Institute of Medical Informatics, Statistics and Documentation, Medical University of Graz, Austria.
49. Interfaculty Institute for Genetics and Functional Genomics, University Medicine Greifswald, Friedrich-Ludwig-Jahn-Str. 15a, 17475 Greifswald, Germany.
50. Department of Nutrition, Harvard School of Public Health and Channing Division of Network Medicine, Brigham and Women's Hospital, USA.
51. German Center for Diabetes Research (DZD), Neuherberg, Germany.
52. Department of Pediatrics, Faculty of Medicine and Life Sciences, University of Tampere, Tampere 33014, Finland.
53. Unit Chronic Disease Epidemiology, Swiss Tropical and Public Health Institute, Basel, Switzerland.
54. University of Basel, Switzerland.
55. Department of Clinical Physiology, Tampere University Hospital, Tampere 33521, Finland.
56. Department of Clinical Physiology, Faculty of Medicine and Life Sciences, University of Tampere, Tampere 33014, Finland.
57. Deutsches Herzzentrum München, Technische Universität München, Munich, Germany.
58. DZHK (German Centre for Cardiovascular Research), partner site Munich Heart Alliance, Munich, Germany.
59. Department of Internal Medicine II - Cardiology, University of Ulm Medical Center, Ulm, Germany.
60. University Medical Centre Mannheim, 5th Department of Medicine, University of Heidelberg, Theodor Kutzer Ufer 1-3, 68167 Mannheim, Germany.
61. Institute of Environmental Medicine, Karolinska Institute, Stockholm, Sweden.
62. Department of Clinical Chemistry, Fimlab Laboratories, Tampere 33520, Finland.
63. Department of Clinical Chemistry, Faculty of Medicine and Life Sciences, University of Tampere, Tampere 33014, Finland.
64. University Medical Center Groningen, University of Groningen, The Netherlands.
65. The Mindich Child Health Development Institute, Icahn School of Medicine at Mount Sinai, New York City, USA.
66. Institute of Human Genetics, Helmholtz Zentrum München, German Research Center for Environmental Health, Neuherberg, Germany.
67. Institute of Human Genetics, Technische Universität München, Munich, Germany.
68. Inserm U1018, University Paris-Sud, UVSQ, University Paris-Saclay, Villejuif, France.
69. Centre for Vision Research, Department of Ophthalmology and Westmead Institute for Medical Research, University of Sydney C24, NSW, 2145, Australia.
70. Institute of Clinical Chemistry and Laboratory Medicine-University Medicine Greifswald, Ferdinand-Sauerbruch-Str., 17475 Greifswald, Germany.
71. DZHK (German Center for Cardiovascular Research), partner site Greifswald, Greifswald, Germany.
72. Department of Psychiatry, University of Groningen, University Medical Center Groningen, P.O. box 30.001, 9700 RB, Groningen, The Netherlands.

73. Department of Psychiatry, Vrije Universiteit, VU University Medical Center, NESDA, A.J. Ernststraat 1187, 1081HL Amsterdam, The Netherlands.
74. Department of Clinical Physiology and Nuclear Medicine, Turku University Hospital, Turku 20521, Finland.
75. Research Centre of Applied and Preventive Cardiovascular Medicine, University of Turku, Turku 20520, Finland.
76. Institute of Physiology, University Medicine Greifswald, 17475 Greifswald, Germany.
77. Division of Cardiovascular Medicine, Brigham and Women's Hospital and Harvard Medical School, Boston MA 02115, USA.
78. Department of Internal Medicine, Erasmus Medical Center, Rotterdam, The Netherlands.
79. Joslin Diabetes Center. Harvard Medical School, Boston, MA, USA.
80. Institute of Molecular Biology and Biochemistry, Centre for Molecular Medicine, Medical University of Graz, Austria.
81. School of Biomedical Sciences and Pharmacy, University of Newcastle, Australia.
82. Molecular Medicine, Pathology North Ph. 0409926764, Newcastle, Australia.
83. Clinic for Internal Medicine A, University Medicine Greifswald, Ferdinand-Sauerbruch-Str., 17475 Greifswald, Germany.
84. Institute of Medical Informatics, Biometry and Epidemiology, Chair of Genetic Epidemiology, Ludwig-Maximilians-Universität, Munich, Germany.
85. Division of Medicine, Turku University Hospital, Turku 20521, Finland.
86. Department of Medicine, University of Turku, Turku 20520, Finland.
87. Department of Internal Medicine, Lausanne University Hospital (CHUV), Lausanne, Switzerland.
88. DZD (German Center for Diabetes Research), Site Greifswald, Greifswald, Germany.
89. Department of Biostatistics, Boston University School of Public Health, 715 Albany Street, Boston, MA 02118, USA.
90. Division of Genetics, Brigham and Women's Hospital and Harvard Medical School, Boston MA.
91. Broad Institute of MIT and Harvard, Cambridge MA 02142 USA.
92. Weis Center for Research, Geisinger Clinic, Danville, Pennsylvania, USA.
93. Department of Epidemiology, Johns Hopkins Bloomberg School of Public Health, Baltimore, USA.

Contents

Supplementary Figures

Supplementary Figure 1. Quantile-Quantile plot of observed versus expected $-\log_{10}$(p-values) of the 1000 Genomes meta-analysis of eGFRcrea 6

Supplementary Figure 2. Novel overlapping meta gene sets 7

Supplementary Figure 3. Regional association plots of the 8 loci with potentially independent association signal..8

Supplementary Tables

Supplementary Table 1. Study information 11

Supplementary Table 2. Characteristics of study subjects 14

Supplementary Table 3. Genotyping and imputation information..15

Supplementary Table 4. Statistical analyses performed by participating studies 18

Supplementary Table 5. Number of variants in the 1000 Genomes and the HapMap Meta-analysis 20

Supplementary Table 6. Gene biology of the genes included in the newly identified loci...........21

Supplementary Table 7. 1000 Genomes lead variants confirming the 39 known loci 23

Supplementary Table 8. Summary statistics of the 14 previously published loci that were not genome-wide significant in the 1000 Genomes meta-analysis. 24

Supplementary Table 9. The four lead variants in novel loci identified by the 1000 Genome meta-analysis that were also available in the HapMap panel 25

Supplementary Table 10. Novel reconstituted gene sets from the DEPICT pathway analysis.....26

Supplementary Table 11. Summary of the 8 independent association signals suggested by joint conditional analysis on reported variants with GCTA...28

Supplementary Table 12. Characterization of variants with smallest p-value in independent association signals identified by the joint conditional analysis 29

Supplementary Table 13. Gene biology of the lead variants in the additional signals.................31

Supplementary Table 14. Polygenic risk score analysis in the TRAILS study 33

Supplementary Table 15. Expression quantitative trait loci (eQTL) lookup 34

Study specific acknowledgements and funding sources for participating studies 35

References...42

Supplementary Figure 1. Quantile-Quantile plot of observed versus expected $-\log_{10}$(p-values) of the 1000 Genomes meta-analysis of eGFRcrea. Shown are p-values for all variants with median imputation quality ≥ 0.4 and with data on ≥ 50% of the available subjects (i.e. ≥ 55,260). P-values were corrected for inflation at study and meta-analysis level, if the genomic control factor $\lambda > 1$. λ_{all} represents the genomic control factor for all variants, λ_{new} considers all variants except those contained in the 53 known loci (published lead variant ± 1MB). Variants considered for λ_{new} were then subdivided into variants with minor allele frequency (MAF) >5% ($\lambda_{NewCommons}$), 0.5% ≤ MAF ≤ 5% ($\lambda_{NewLessCommons}$) and MAF < 0.5% ($\lambda_{NewRares}$).

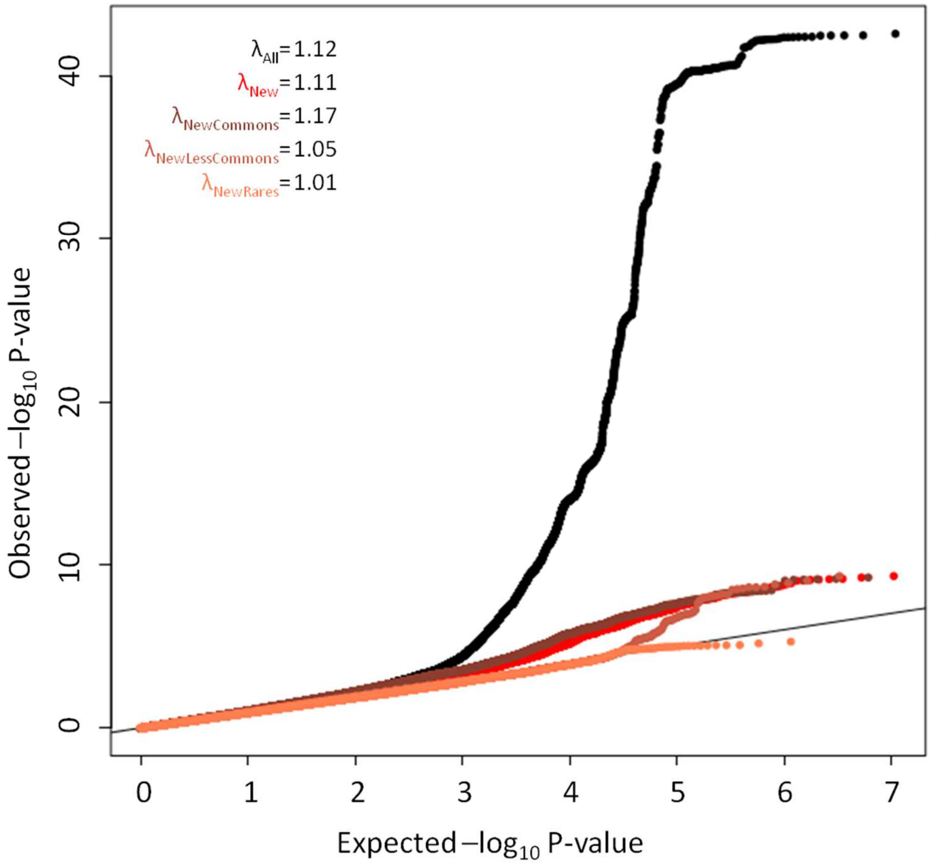

Supplementary Figure 2. Novel overlapping meta gene sets. Shown are the 20 novel meta gene sets, based on the DEPICT analysis of 9,270 variants from the HapMap and 1000G meta-analysis. The coloring of the meta gene sets represents the smallest p-value of all comprised gene sets and is coded on a continuous scale. The overlap between meta gene sets was estimated by computing the pairwise Pearson correlation coefficient ρ between each pair of gene sets followed by a ranking: 0.3 ≤ ρ ≤ 0.5, low overlap; 0.5 < ρ < 0.7, medium overlap; ρ ≥ 0.7, high overlap. Overlap is shown by edges between gene set nodes; edges representing overlap corresponding to ρ ≤ 0.3 are not shown. The network was drawn with Cytoscape (http://cytoscape.org/).

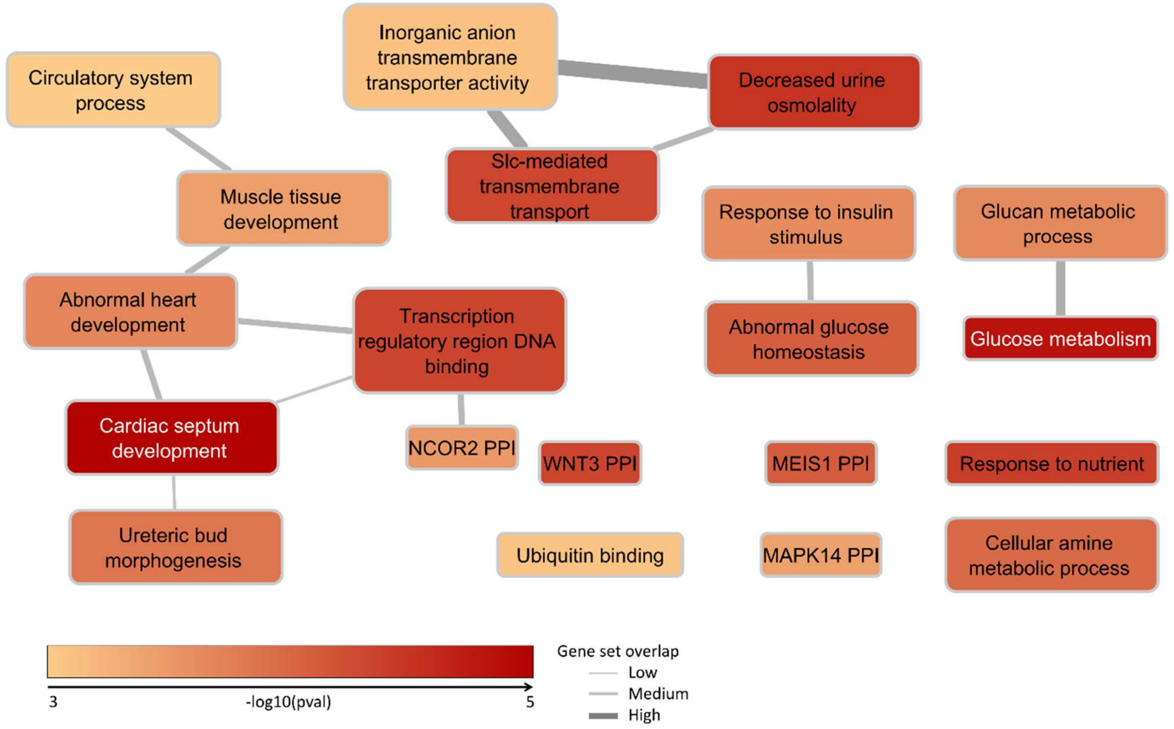

Supplementary Figure 3. Regional association plots of the 8 loci with potentially independent association signal. Shown are the p-values (on a $-\log_{10}$ scale) versus genomic position (on GRCh build 37) in the 1000 Genomes meta-analysis before ("Unconditional" left panels: **A1-H1**) and after conditioning on the reported variant using the GCTA approach ("Conditional" right panels: **A2-H2**). The reported variant is highlighted in blue and the potentially independent association signal is highlighted in red. The red horizontal line indicates the genome-wide significance threshold of $5x10^{-8}$.

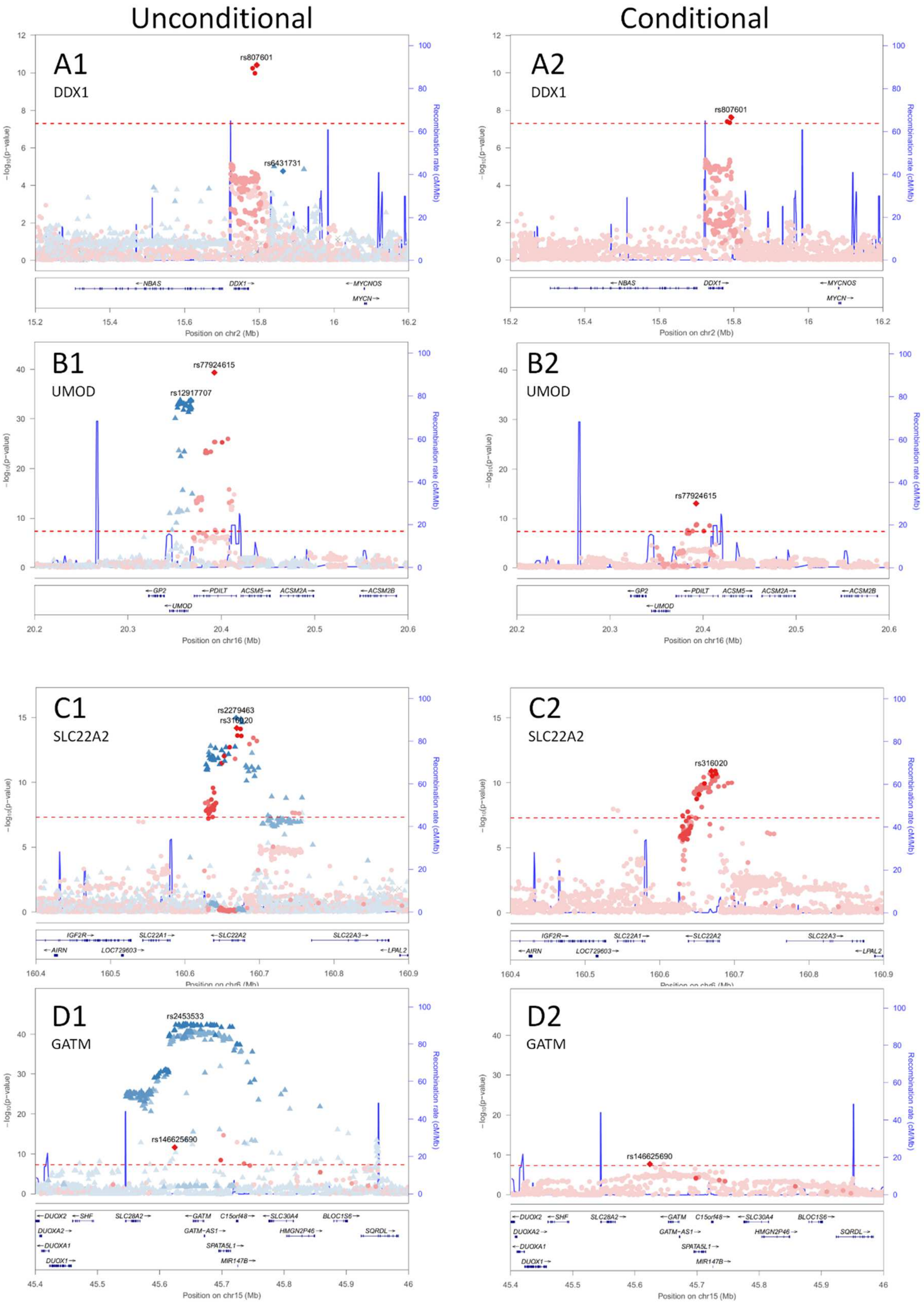
Unconditional
Conditional
A1
DDX1
rs807601
rs6431731
A2
DDX1
rs807601
NBAS
DDX1
MYCNOS
MYCN
Position on chr2 (Mb)
B1
UMOD
rs77924615
rs12917707
B2
UMOD
rs77924615
GP2
PDILT
ACSM5
ACSM2A
ACSM2B
UMOD
Position on chr16 (Mb)
C1
SLC22A2
rs2279463
rs316020
C2
SLC22A2
rs316020
IGF2R
SLC22A1
SLC22A2
SLC22A3
AIRN
LOC729603
LPAL2
D1
GATM
rs2453533
rs146625690
D2
GATM
rs146625690
DUOX2
SHF
SLC28A2
GATM
C15orf48
SLC30A4
BLOC1S6
DUOXA2
GATM-AS1
HMGN2P46
SQRDL
DUOXA1
SPATA5L1
DUOX1
MIR147B
Position on chr15 (Mb)
−log10(p−value)
Recombination rate (cM/Mb)

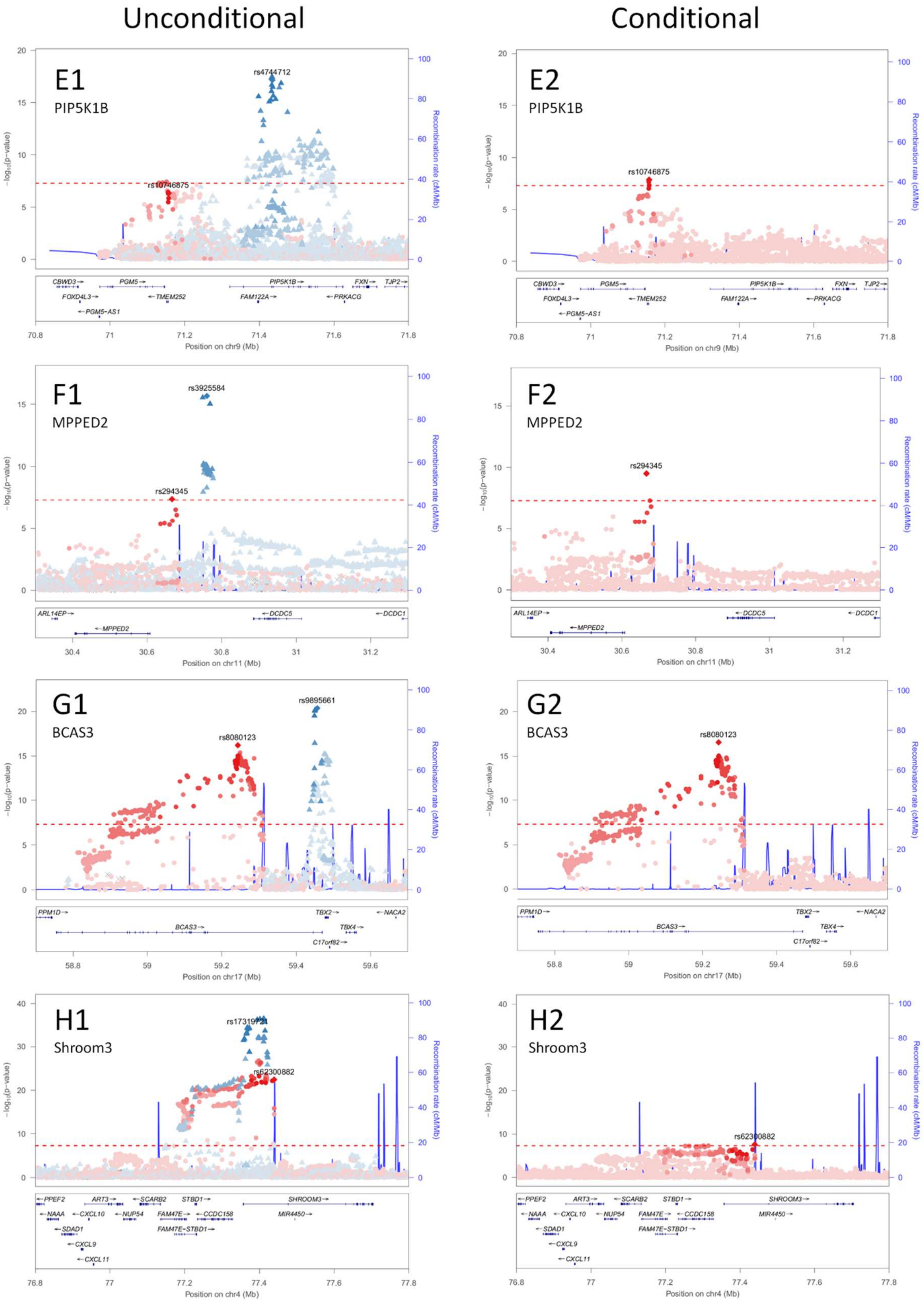

Unconditional
Conditional
E1
PIP5K1B
rs4744712
rs10746875
E2
PIP5K1B
rs10746875
Position on chr9 (Mb)
F1
MPPED2
rs3925584
rs294345
F2
MPPED2
rs294345
Position on chr11 (Mb)
G1
BCAS3
rs9895661
rs8080123
G2
BCAS3
rs8080123
Position on chr17 (Mb)
H1
Shroom3
rs17319721
rs62300882
H2
Shroom3
rs62300882
Position on chr4 (Mb)
-log10(p-value)
Recombination rate (cM/Mb)

Supplementary Table 1. Study information: Full study names are reported in the Acknowledgements section.

Study	Study Design	Study exclusions	Creatinine Measurement	Cystatin measurement
3C[1,2]	Prospective population-based	None.	Modified kinetic Jaffe reaction.	Particle-enhanced immuno-nephelometric method (BNII, Dade-Behring/ Siemens)
AGES[3]	Population based	We excluded subjects with sample failure, genotype mismatch with reference panel and sex mismatch.	Jaffé reaction.	NA
ARIC[4]	Prospective, population-based	Of all genotyped individuals of European ancestry, we excluded individuals based on discrepancies with previous genotypes, disagreement between reported and genotypic sex, one randomly selected member of a pair of first-degree relatives, or outlier based on measures of average DST or >8 SD away on any of the first 10 principal components.	Modified kinetic Jaffé reaction.	Particle enhanced immunonephelometric assay (N Latex Cystatin C, Dade Behring).
ASPS[5,6]	Prospective study	We excluded subjects with history of neuropsychiatric disease, previous stroke and/or TIA, and dementia. Of the participants who underwent genotyping, we made the following exclusions: sample call rate <98% (74). This resulted in a total of 848 genotyped individuals.	Modified kinetic Jaffé reaction.	NA
BMES[7-9]	Prospective cohort study	We excluded subjects with sample call rate <95%, outlying autosomal heterozygosity, sex discrepancies or ambiguous sample identification, cryptic relatedness (average IBD sharing proportion > 0.1875), non-European ancestry.	Measured within 4 hours of collection using a Hitachi 747 Biochemistry analyzer (Roche reagents, modified kinetic Jaffé).	NA
CoLaus[10]	Population-based	We excluded subjects with call rate <90% and related individuals	Serum creatinine was measured by the Jaffe kinetic compensated method (2.9% – 0.7% maximum inter and intra-batch CVs) on fasting samples.	NA
EGCUT1, EGCUT 2[11]	Population-based	We excluded subjects with missing creatinine levels; genetic outliers; cryptic relatedness (one random member up to 2nd cousins was only included)	Modified Jaffé protein compensated method in the serum.	NA
FamHS[12]	Family based	We excluded subjects with age <18, call rate <98%, pHWE <10x10^{-6}, sex mismatch and subjects of non-European ancestry.	Thin film adaptation of the amidohydrolase enzymatic method using the Vitros analyzer (Johnson & Johnson Clinical Diagnostics, Inc. Rochester NY 14650).	Immune particle-enhanced turbidimetric (PET) kit (DAKO A/S, Produktionsvej 42, DK-2600 Glostrup, Denmark. Code no. K0071)
FHS[13-15]	Prospective family-based	We excluded subjects with sample call rate <97%, genotype heterozygosity > 5 SDs, and ambiguous family data.	Modified kinetic Jaffé reaction.	Particle-enhanced immunonephelometric method, Cystatin C was measured (BNII, Dade-Behring).
GENDIAN[16,17]	Cohort study of T2D complications	We excluded subjects with ESRD or advanced, histologically proven diabetic nephropathy or missing phenotype, subjects with call-rate<95%, related and duplicated subjects, subjects with gender mismatch and non-European subjects.	Enzymatic assay.	Dade Behring assay (BNII)
HABC	Prospective cohort study	We excluded subjects with sample failure, genotypic sex mismatch and first-degree relative of an included individual based on genotype data.	Colorimetric technique on a Johnson & Johnson VITROS 950 Chemistry Analyzer (Johnson & Johnson, New Brunswick, N.J., USA) using the enzymatic method.	BNII nephelometer (Dade Behring Inc., Deerfield, Ill., USA) that utilized a particle enhanced immunonephelometric assay (N Latex Cystatin C).

HCS[18]	Population-based	We excluded subjects with genotype call rate <0.95, outlying heterozygosity, gender discrepancies, missing clinical data, cryptic relationships, non-European ancestry or missing creatinine measurement.	Siemens Dimension Vista 1500 Intelligent Lab System using a modified Jaffé assay in a NATA accredited lab.	NA
HPFS[19-23]	Nested case-control study of T2D	None.	Modified kinetic Jaffé reaction in plasma.	NA
INGI-CARLANTINO[24-26]	Isolated population	We excluded subjects with call rate <97%.	Jaffé reaction.	NA
CILENTO[27-34]	Cross-sectional population-based study of isolated populations with pedigree information	We excluded subjects with age <20 years.	Modified kinetic Jaffé reaction.	NA
INGI-FVG[24-26]	Isolated population	We excluded subjects with call rate <97%.	Jaffé reaction.	NA
INGI-VAL BORBERA[35]	Family Population-based	We excluded subjects with call rate <95%.	Jaffé reaction.	NA
IPM I + IPM II[36]	Hospital-based	None.	Colorimetric method in (CPT82565) performed by the New York State / CLIA Clinical Chemistry Laboratory at Mount Sinai Medical Center.	NA
KORA-F3[37,38]	Prospective population based	None.	Modified kinetic Jaffe reaction.	Particle-enhanced immunonephelometric method (BNII, Dade-Behring).
KORA-F4[37,38]	Prospective population based	None.	Modified kinetic Jaffe reaction	Particle-enhanced immunonephelometric method (BNII, Dade-Behring).
Lifelines[39-41]	Prospective population based	None.	enzymatic assay, IDMS traceable (Roche, Mannheim, Germany)	NA
MESA[42]	Community-based cohort study	None.	Serum creatinine was measured by rate reflectance spectrophotometry using thin film adaptation of the creatine amidinohydrolase method on the Vitros analyzer (Johnson & Johnson Clinical Diagnostics, Inc., Rochester, NY 14650). The laboratory analytical CV is 2.2%. All creatinine measurements for the MDRD Study were performed at Cleveland Clinic Labs using a CX3 assay. The Vitros analyzer used here was previously calibrated to a CX3 machine with the Cleveland Clinic lab and found the results were nearly identical.	BNII nephelometer (Dade Behring Inc., Deerfield, IL) that utilizes a particle enhanced immunonepholometric assay (N Latex Cystatin-C) 7 on fasting plasma specimens stored at -70°C. The assay is stable over 5 cycles of freeze / thaw. Among 61 healthy individuals with 3 cystatin-C measurements over a 6-month period, the intra-individual coefficient of variation was 7.7%.
MICROS[43,44]	Cross-sectional, population-based study on extended pedigrees	We excluded subjects with call rate <95%, showing excess of heterozygosity, or being classified as outliers by IBS clustering analysis.	Enzymatic photometric assay using an ADVIA1650 clinical chemistry analyzer (Siemens Healthcare Diagnostics GmbH, Eschborn, Germany).	BN-ProSpec analyzer (Dade Behring, Marburg, Germany) at the Institute for Clinical Chemistry and Laboratory Medicine, Regensburg University Medical Center, Germany.
NHS[19,21,22,45-47]	Nested case-control study of T2D	None.	Modified kinetic Jaffé reaction in plasma. Creatinine values were not normalized to the Cleveland Clinic standard.	NA

NSPHS[48,49]	Cross-sectional, family-based	We excluded subjects with call rate <97%, mismatch between reported and genotypic sex, unexpectedly low genomic sharing with first degree relatives, excess of autosomal heterozygosity, or outliers identified by IBS clustering analysis.	Enzymatic photometric assay in plasma using an ADVIA1650 clinical chemistry analyzer (Siemens Healthcare Diagnostics GmbH, Eschborn, Germany).	NA
Rotterdam Study I [50-53]	Prospective population based study	We excluded subjects with call rate < 97.5%, excess autosomal heterozygosity >0.336 (~FDR <0.1%), mismatch between called and phenotypic gender, or outliers identified by the IBS clustering analysis with >3 SDs from population mean or IBS probabilities >97%.	Modified kinetic Jaffé reaction.	NA
SAPALDIA	Population based	We excluded subjects with cryptic relatedness and call rate <95%.	Jaffé reaction (Roche) and calibrated to the Roche enzymatic gold standard reference yielding slightly lower serum creatinine measurements than the Cleveland Clinic Jaffé reaction.	NA
SHIP[54,55]	Prospective population-based	We excluded subjects with call rate <92%, duplicate samples (by IBS estimation) and individuals with reported or genotypic gender mismatch.	Jaffé method. (A blood sample was drawn from the cubital vein in the supine position - the participants were non-fasting due to the duration of the cumulative examinations, 4-6 hours in total).	Siemens N Latex Cystatin C assay, a particle-enhanced nephelometric immunoassay, on the BN ProSpec® System.
SHIP-TREND	Prospective population-based	We excluded subjects with no genotype, known T2D, call rate <94%, duplicate samples (by IBS estimation), individuals with reported or genotypic gender mismatch.	Jaffé method.	Dimension Vista® System, CYSC Flex® reagent cartridge, SIEMENS, Eschborn, Germany
WGHS[56]	Prospective population based	We excluded subjects of non-European ancestry and with call rate < 98%.	Rate-blanked method based on the Jaffé reaction using Roche Diagnostics reagents with reproducibility of 3.67% and 1.60% at concentrations of 1.17 and 6.40 mg/dL, respectively.	NA
YFS	Population based	None.	Jaffé method (picric acid; Olympus Diagnostica GmbH) from frozen plasma samples in 2007.	NA

Abbreviations: T2D=type 2 diabetes.

Supplementary Table 2. Characteristics of study subjects. Shown are the number of subjects in the meta-analysis and the descriptive statistics of sex, age, eGFRcrea and eGFRcys per study.

Study	Sample Size eGFRcrea	Sample Size eGFRcys	Women % (n)	Age: mean (SD), years	eGFRcrea: mean (SD), ml/min/1.73 m^2	eGFRcys: mean (SD), ml/min/1.73 m^2
3C	6,431	NA	60.8 (3,911)	74.3 (5.5)	73.1 (16.9)	NA
AGES	3,219	NA	58.0 (1,867)	76.0 (5.0)	73.0 (20.0)	NA
ARIC	9,038	7,151	53.0 (4,788)	54.3 (5.7)	89.8 (18.0)	84.3 (19.7)
ASPS	829	NA	56.7 (470)	65.5 (8.0)	80.4 (20.3)	NA
BMES	2,437	NA	56.8 (1,385)	69.4 (9.5)	78.7 (20.2)	NA
CILENTO	1,092	NA	54.6 (596)	53.1 (18.0)	89.5 (21.9)	NA
COLAUS	5,409	NA	53.0 (2,863)	53.4 (10.8)	83.2 (16.4)	NA
EGCUT1	4,437	1,037	56.5 (2,509)	51.6 (19.0)	97.4 (28.0)	85.5 (16.9)
EGCUT2	1,018	NA	51.0 (520)	39.1 (15.3)	115.7 (26.3)	NA
FamHS	3,838	521	52.4 (2,012)	52.1 (13.7)	91.6 (20.1)	86.3 (33.5)
FHS	3,051	2,992	53.3 (1,626)	61.0 (9.5)	84.8 (19.1)	83.9 (17.7)
GENDIAN	450	532	47.1 (250)	60.8 (11.0)	70.0 (20.2)	85.3 (27.1)
HABC	1,661	1,661	47.1 (784)	74.0 (3.0)	71.2 (14.8)	77.0 (19.9)
HCS	2,113	NA	50.0 (1,056)	66.3 (7.7)	80.1 (18.5)	NA
HPFS	818	NA	0 (0)	64.7 (8.3)	85.2 (11.7)	NA
INGI-CARLANTINO	412	NA	59.5 (245)	50.1 (16.3)	93.9 (21.7)	NA
INGI-FVG	848	NA	59.1 (501)	52.5 (16.6)	90.7 (21.9)	NA
INGI-VAL BORBERA	1,754	NA	56.0 (983)	55.6 (17.6)	87.4 (21.1)	NA
IPM I	440	NA	30.2(133)	62.3 (13.3)	94.7 (36.7)	NA
IPM II	1,307	NA	48.6 (635)	67.6 (9.2)	86.0 (27.7)	NA
KORA-F3	3,095	1,642	51.3 (1,587)	57.1 (12.9)	88.1 (21.5)	111.8 (26.6)
KORA-F4	2,936	1,811	51.6 (1,514)	56.2 (13.2)	88.2 (21.5)	109.8 (22.8)
LIFELINES	13,386	NA	58.3 (7,795)	48.8 (11.4)	90.1 (16.3)	NA
MESA	2,520	2,520	52.0 (1,311)	63.0 (10.0)	82.4 (18.3)	90.0 (21.7)
MICROS	1,185	NA	56.5 (678)	46.2 (16.1)	94.6 (20.9)	NA
NHS	786	NA	100 (786)	59.5 (6.5)	86.2 (22.1)	NA
NSPHS	563	NA	53.1 (300)	51.7 (18.3)	91.0 (22.1)	NA
RS I	4,595	NA	61.7(2,835)	70.0 (9.0)	77.2 (17.3)	NA
SAPALDIA	1,444	NA	51.0 (737)	52.3 (11.2)	90.3 (17.3)	NA
SHIP	3,210	3,210	51.8 (1,663)	54.5 (15.3)	90.4 (23.6)	97.1 (25.4)
SHIP-TREND	986	986	56.2 (554)	50.1 (13.7)	92.4 (22.1)	122.1 (22.1)
WGHS	23,186	NA	100.0 (23,186)	54.7 (7.1)	84.2 (20.6)	NA
YFS	2,023	NA	54.7 (1,107)	37.6 (5.0)	100.5 (15.9)	NA
**TOTAL**	**110,517**	**24,063**				

SD=standard deviation.

Supplementary Table 3. Genotyping and imputation information. Shown are the details of the genotyping and imputation procedures per study.

Study name	Array type	Genotype calling	QC filters for genotyped SNPs used for imputation	No of SNPs used for imputation	Pre-phasing Software	Imputation Software	Reference panel	Filtering of imputed genotypes	Type of reported imputation quality*
3C	Illumina Human610 Quad BeadChip	Illumina	pHWE<1e-6, call rate < 98 %, MAF < 0.01, SNPs not successfully mapped to build 37	521,648	SHAPEIT v1	IMPUTE v2.2.2	1000 Genomes Phase I Version 3 All	none	IMPUTE2 Info
AGES	Illumina Hu370CNV	Illumina BeadStudio	pHWE<1e-6, call rate<97%, mishap p<1e-9, MAF<0.01, SNPs not in HapMap, remove G/CA/T SNPs	324,603	MACH v1.0.16	minimac 10.3.12	GIANT 1000 Genomes Phase I Version 3 All	none	minimac Rsq
ARIC	Affymetrix 6.0	Birdseed	call rate <95%, MAF<0.5%, pHWE<10e-5	682,749	SHAPEIT	IMPUTE2	1000 Genomes Phase I Version 3 All	none	IMPUTE2 Info
ASPS	Illumina Human610-Quad BeadChip	Illumina	pHWE<1e-6, call rate<98%, MAF<0.01	536,954	SHAPEIT v1	IMPUTE2	1000 Genomes Phase I Version 3 All	MAF <=0.01, MAF >=0.99, IMPUTE2 info <=0.3	IMPUTE2 Info
BMES	Illumina 670K-Quad	Illumina	call rate <97%, pHWE<10E-4, MAF<0.01, SNPs not in reference or stranded diverging from 1,356 samples independently genotyped for the Illumina 610K array	513,270	MACH v1.0.18	minimac 2012.5.29	1000 Genomes Phase I Version 3 Eur	r2>0.04	minimac Rsq
CoLaus	Affymetrix 500K	Affymetrix	pHWE<1e-7, call rate<90%, MAF<0.005, SNPs without rs number	388,663	MACH v1.0.16	minimac 10.3.12	1000 Genomes Phase I Version 3 All	none	minimac Rsq
EGCUT1	Illumina OmniExpress	Genome Studio	Sample call rate 0,95; SNP call rate 0,95; pHWE<1e-6; MAF <0.01	615,575	SHAPEIT v1	IMPUTE version 2.2.2	1000 Genomes Phase I Version 3 All	none	SNPTEST info
EGCUT2	Illumina Human370CNV	Genome Studio	Sample call rate 0,95; SNP call rate 0,95; pHWE<1e-6; MAF <0.01	309 389	SHAPEIT v1	IMPUTE version 2.2.2	1000 Genomes Phase I Version 3 All	none	SNPTEST info
Family Heart Study	ILLUMINA 550K, ILLUMINA 610K ILLUMINA 1M	BeadStudio -gencall v3.0	call rate <98%, pHWE<10E-6, MAF<1%	519,261	MACH v1.0.16	minimac 10.3.12	1000 Genomes Phase I Version 3 All	none	minimac Rsq
FHS	Affymetrix 500K Affymetrix 50K supplemental	Affymetrix	pHWE<1e-6, call rate<97%, mishap p<1e-9, MAF<0.01, Mendelian errors>100, SNPs not in Hapmap or strandedness issues merging with Hapmap	378,163	MACH v1.0.16	minimac 10.3.12	GIANT 1000 Genomes Phase I Version 3 All	none	minimac Rsq
Gendian	Genome-Wide Human SNP Array 6.0	Birdseed	pHWE < 10-6; monomorphic SNPs; MAF>.1 & call rate<.9 MAF>.0x & callrate1-.0x	747,402	MACH v1.0.18.c	minimac 2012.10.09	GIANT 1000 Genomes Phase I Version 3 All	none	minimac Rsq
HABC	Illumina Human 1M-Duov3	Beadstudio-Gencall v3.0	Call rate < 98%; pHWE<1e-6; Mendelian errors; Exclude Duplicate samples, sex mismatch	914,263	MACH version 1.0.16	minimac 10.3.12	1000 Genomes Phase I Version 1 All	none	minimac Rsq

HCS	Affymetrix Kaiser Axiom	Affymetrix	call rate <97%, pHWE<10E-4, MAF<0.01, SNPs not in 1000 Genomes or strandedness issues with merging with 1000 Genomes.	517,693	MACH v1.0.18	minimac 2012.5.29	1000 Genomes Phase I Version 3 Eur	r2>0.04	minimac Rsq
HPFS	Affymetrix Genome-Wide Human 6.0 array	Birdseed	call rate <97%, pHWE<10E-4, MAF <0.02, >1 discordance/12 replicates, significant plate associations	672,833	MACH v1.0.16	minimac 10.3.12	1000 Genomes Phase I Version 3 All	MAF=0	minimac Rsq
Cilento	Illumina 370 K (859 individuals) , Illumina OmniExpress 700K (288 individuals)	Illumina	Imputation was performed in the two groups (859 and 288 individuals) separately, using the following filters: call rate<95%, MAF<1%. For the directly typed SNPs in common between the two groups, the real genotype was used in the association analysis, while the imputation dosage was considered for the other SNPs.	306,995 (859 subjects) 588,083 (288 subjects)	MACH v1.0.16	minimac 2012.05.29	GIANT 1000 Genomes Phase I Version 3 All	only monomorphic SNPs were excluded from the analysis	minimac Rsq
INGI CARLAN TINO	Illumina 370K	Genome Studio Illumina	call rate<97%, MAF<0.01,pHWE<0.00001	310,162	SHAPEIT2	IMPUTE2	1000 Genomes Phase I Version 3 All	MAF<0.05, imputation quality=0.4	IMPUTE2 Info
INGI-FVG	Illumina 370K	Genome Studio Illumina	call rate<97%, MAF<0.01,pHWE<0.00001	337,266	SHAPEIT2	IMPUTE2	1000 Genomes Phase I Version 3 All	MAF<0.05, imputation quality=0.4	IMPUTE2 Info
INGI VAL BORBER A	Illumina 370K	Genome Studio Illumina	call rate<97%, MAF<0.01,pHWE<0.00001	337,266	SHAPEIT2	IMPUTE2	1000 Genomes Phase I Version 3 All	r2>0.04	IMPUTE2 Info
IPM I	Affymetrix 6.0	Birdseed	sample call rate<0.95, SNP call rate<0.95, pHWE<1E-4, MAF<0.01	711,270	SHAPEIT	IMPUTE2	1000 Genomes Phase I Version 3 All	none	IMPUTE2 Info
IPM II	Illumina HumanOmniExpr essExome-8v1	Genome Studio	sample call rate<0.99, SNP call rate<0.95, pHWE<5E-5, no minor allele	865,711	SHAPEIT v2	IMPUTE2	1000 Genomes Phase I Version 3 All	none	IMPUTE2 Info
KORA-F3	HumanOmniExpr ess 12v1 Illumina HumanOmni 2.5-4	Genome Studio 2010.3 Genome Studio 2011.1	call rate > 98% pHWE <5*10-6 MAF > 0.01 only SNPs that were genotyped with good quality on both chips	588,307	SHAPEIT v2	IMPUTE v2.3.0	1000 Genomes Phase I Version 3 All	none	IMPUTE2 Info
KORA-F4	Affymetrix Axiom	Affymetrix	call rate > 98% pHWE <5*10-6 MAF > 0.01	508,532	SHAPEIT v2	IMPUTE v2.3.0	1000 Genomes Phase I Version 3 All	none	IMPUTE2 Info
Lifelines	Illumina Cyto SNP12 v2	Genome Studio	SNPs with call rate < 95%, pHWE < 0.001, MAF < 0.01, samples with excess heterozygosity or non-Caucasian origin	257,581	SHAPEIT v2	minimac	1000 Genomes Phase I Version 3 All	none	PLINK

MESA	Affymetrix Genome-Wide Human SNP Array 6.0	Birdseed	call rate≥95%, MAF>1%, observed heterozygosity ≤ 53%	897,979	BEAGLE	IMPUTE v2.2.2	1000 Genomes Phase I Version 3 All	None	IMPUTE2 Info
Micros	Illumina 317K, Illumina 370K, Illumina 550K	BeadStudio	identical (non-twins) samples excluded, individual call rate 0.98, SNP call rate > 0.98, MAF > 0.001, heterozygosity check, sex inconsistency check	303,859	Mach v1.0.16.c	minimac	GIANT 1000 Genomes Phase I Version 3 All	none	minimac Rsq
NHS	Affymetrix Genome-Wide Human 6.0 array	Birdseed	call rate <97%, pHWE<10E-4, MAF <0.02, >1 discordance/12 replicates, significant plate associations	672,396	MACH v1.0.16	minimac 10.3.12	1000 Genomes Phase I Version 3 All	MAF=0	minimac Rsq
NSPHS	Illumina Infinium HapMap300v2	BeadStudio	Genotyping call rate >95% subject call rate >90%, minor allele frequency (MAF) >0.01, pHWE < 3.4 x 10e-8.	306,086	IMPUTE2	IMPUTE2	1000 Genomes Phase I Version 3 All	none	IMPUTE2 Info
Rotterdam Study I	Illumina v3 Infinium II HumanHap550	BeadStudio	pHWE<1e-6, call rate<98%, MAF<0.01, Mendelian errors>100,	512,849		MACH	GIANT 1000 Genomes Phase I Version 3 All	None	MACH Rsq
SAPALDIA	Illumina Human 610 Quad BeadChip	Gencall	MAF < 0.01, call rate<=95%, pHWE<1e-6	545,131	Mach v1.0.16.a	minimac 2012.05.29	GIANT 1000 Genomes Phase I Version 3 All	none	minimac Rsq
SHIP	Affymetrix Genome-Wide SNP 6.0	Birdseed	pHWE <=0.0001, call rate <=0.8	905,910	IMPUTE v2.1.2.3	IMPUTE v2.2.2	1000 Genomes Phase I Version 3 All	No monomorphic markers	IMPUTE2 Info
SHIP-TREND	Illumina Human Omni 2.5	Illumina GenCall	pHWE <=0.0001, call rate <=0.9, monomorphic SNPs	1,824,743	IMPUTE v2.1.2.3	IMPUTE v2.2.2	1000 Genomes Phase I Version 3 All	No monomorphic markers	IMPUTE2 Info
INGI-Val Borbera	Illumina 370k (1664 subjects) Illumina OmniExpress 700K (121 subjects)	BeadStudio analysis software	call rate >=90%; MAF >=1%; pHWE p <=0.001	332,887 (1,664 subjects) 648,130 (121 subjects)	SHAPEIT2 (1,664 subjects) none (121 subjects)	IMPUTE2	1000 Genomes Phase I Version 3 All	none	IMPUTE2 Info
WGHS	Illumina HumanHap Duo and iSelect	BeadStudio v3.3	pHWE>1e-6, call rate>90%, no MAF restriction	332,927	MACH v1.0.16	minimac 2012.5.29	1000 Genomes Phase I Version 3 All	none	minimac Rsq
YFS	Illumina 670k custom	Illuminus	pHWE<1e-6, call rate<95%, MAF<0.01, heterozygosity	546,677	SHAPEIT v1	IMPUTE v2.1.2	1000 Genomes Phase I Version 3 All	none	SNPTEST Info

pHWE=P-value of test for deviation from Hardy–Weinberg equilibrium, MAF=minor allele frequency.

*All but four studies contributed either MACH/ minimac RSQ or ImputeV2 info score: EGCUT1, EGCUT2 and YFS provided SNPTest info; LIFELINES provided Plink imputation quality.

Supplementary Table 4. Statistical analyses performed by participating studies.

Study name	Data management and statistical analysis	Population stratification or genetic Principal Components (PCs)
3C	SAS for residuals calculation, SNPtest v2.4.1 for GWAS using an additive model (-frequentist 1), with a missing data likelihood score test (-method score)	Genomic control factor lambda was 0.983 and 0.949 for eGFRcrea and eGFRcys analyses, respectively. Consequently, analyses were not adjusted for PCs.
AGES	R, ProbABEL	While no significant stratification was observed, the first two PCs were included as covariates in the analysis.
ARIC	SNPTEST v2	The first 10 PCs were included in the analysis.
ASPS	SPSS for calculation of residuals, PLINK for GWAS	Neither population stratification nor PC adjustment was applied.
BMES	SAS v9.3	Analyses were adjusted for the first 2 PCs estimated using Eigenstrat.
CoLaus	Matlab	It was observed significant association between eGFRcrea and the first four PCs. Therefore, the first four PCs were included in the analysis.
EGCUT1	R; PLINK 1.07; SNPTEST 2.4.1	Observations were excluded based on identity-by-state (IBS) clustering using PLINK (PI_HAT >0.10). The first 10 PCs were included in the analysis.
EGCUT2	R; PLINK 1.07; SNPTEST 2.4.1	Observations were excluded based on identity-by-state (IBS) clustering using PLINK (PI_HAT > 0.10). The first 10 PCs were included in the analysis.
FamHS	R, linear mixed effect models and kinship approach to account for relatedness	eGFRcrea: PC7 (r^2=0.0020, p=0.0290) and PC2 (r^2=0.0019, p=0.0359) for men are significant.
FHS	R, linear mixed effect models and GEE models, robust variance option to account for relatedness	Significant association between eGFRcrea and the first 10 PCs was observed therefore, the 10 PCs were included in the analysis.
Gendian	R, SAS, ProbABEL	Checks for European ancestry and population outlier were applied.
HABC	R	Analyses were adjusted for the first PC.
HCS	SAS v9.3	Analyses were adjusted for the first 2 PCs estimated using Eigenstrat.
HPFS	ProbABEL, linear regression	Population structure was investigated by PC analysis[57]. The top 3 eigenvectors were included in the analyses.
Cilento	R, linear model, GenABEL and ProbABEL (mmscore function was used to account for relatedness)	Neither population stratification nor PC adjustment was applied.
INGI-CARLANTINO	R, GenABEL, Grammar	Because of the presence of close relatives, statistical analyses were adjusted for relatedness by including the kinship matrix into a mixed model framework.
INGI-FVG	R, GenABEL, Grammar	Because of the presence of close relatives, statistical analyses were adjusted for relatedness by including the kinship matrix into a mixed model framework.
IPM I	score test in SNPTEST	Outlier samples were identified and exclude based on CEU ancestry as from Hapmap, PC analysis and IBS clustering.
IPM II	score test in SNPTEST	Outlier samples were identified and exclude based on CEU ancestry as from Hapmap, PC analysis and IBS clustering.
KORA-F3	R,SAS, ProbABEL	Samples were checked for European ancestry, population outlier, and comparison with other genotyping of the same individuals.

KORA-F4	R,SAS, ProbABEL	Samples were checked for European ancestry, population outlier, and comparison with other genotyping of the same individuals.
Lifelines	PLINK	Analyses were adjusted for the first 10 PCs.
MESA	PLINK	Analyses were adjusted for the first 3 PCs.
Micros	R, GenABEL	Study village was included as fixed effect. Statistical analyses were adjusted for relatedness by including the kinship matrix into a mixed model framework.
NHS	ProbABEL, linear regression	Population structure was investigated by PCA[57]. The top 3 eigenvectors were included in all CKDGen analyses.
NSPHS	R, GenABEL	Statistical analyses were adjusted for relatedness by including the kinship matrix into a mixed model framework.
Rotterdam Study I	ProbABEL	Analyses were adjusted for the first 5 PCs.
SAPALDIA	ProbABEL	PCs derived using Eigenstrat were included in the analysis model.
SHIP	InterSystems Caché, QUICKTEST v0.95	No population stratification was observed by PC and multidimensional scaling analyses.
SHIP-TREND	InterSystems Caché, QUICKTEST v0.95	No population stratification was observed by PC and multidimensional scaling analyses.
INGI-Val Borbera	R, Genome-wide Efficient Mixed Model Association algorithm (GEMMA) to account for relatedness	No PC adjustment was applied.
WGHS	ProbABEL, R, Unix	Neither population stratification nor PC adjustment was applied.
YFS	SNPTEST v2.4.0	Analyses were adjusted for the first 4 PCs.

Supplementary Table 5. Number of variants in the 1000 Genomes and the HapMap Meta-analysis*. Shown are the numbers and percentages by categories of minor allele frequency (MAF) and imputation quality (IQ). All imputed variants in the 1000 Genomes meta-analysis (10,971,307 variants), all imputed variants in the HapMap meta-analysis (2,433,307 SNPs) and the SNPs in both 1000 Genomes and HapMap meta-analysis (2,408,573 SNPs) are compared in categories of IQ across all MAF bins.

MAF	IQ	All variants in 1000 Genomes meta-analysis	All variants in HapMap meta-analysis	Overlapping variants in 1000 Genomes meta-analysis	Overlapping variants in HapMap meta-analysis
All	0.8 < IQ	8,103,124 (73.86%)	2,249,027 (92.41%)	2,334,834 (96.94%)	2,247,511 (93.31%)
	0.4 < IQ ≤ 0.8	2,836,399 (25.85%)	154,161 (6.33%)	73,657 (3.06%)	147,152 (6.11%)
	IQ ≤ 0.4	31,784 (0.29%)	30,570 (1.26%)	82 (<0.01%)	13,910 (0.58%)
MAF>0.05	0.8 < IQ	5,885,422 (92.53%)	2,057,447 (94.6%)	2,118,463 (97.92%)	2,056,299 (95.04%)
	0.4 < IQ ≤ 0.8	475,160 (7.47%)	103,467 (4.76%)	45,070 (2.08%)	100,472 (4.64%)
	IQ ≤ 0.4	63 (<0.01%)	14,018 (0.64%)	7 (<0.01%)	6,769 (0.31%)
0.01<MAF<=0.05	0.8 < IQ	1,585,176 (62.54%)	191,580 (74.02%)	216,371 (88.3%)	191,212 (78.04%)
	0.4 < IQ ≤ 0.8	946,240 (37.33%)	50,694 (19.59%)	28,587 (11.67%)	46,680 (19.05%)
	IQ ≤ 0.4	3,431 (0.13%)	16,552 (6.4%)	75 (0.03%)	7,141 (2.91%)
MAF<=0.01	0.8 < IQ	632,526 (30.47%)	0 (0%)	0 (0%)	0 (0%)
	0.4 < IQ ≤ 0.8	1,414,999 (68.17%)	0 (0%)	0 (0%)	0 (0%)
	IQ ≤ 0.4	28,290 (1.36%)	0 (0%)	0 (0%)	0 (0%)
Number of variants		10,971,307	2,433,307	2,408,573	2,408,573

Abbreviations: MAF is the minor allele frequency; IQ is the imputation quality metric computed as median of info score (ImputeV2) or RSQ (minimac) across studies.

*Only variants analyzed in at least half of all subjects are counted (i.e. n ≥ 55,260 and n ≥ 66,910 in the 1000 Genomes and Hapmap imputed genotypes, respectively).

Supplementary Table 6. Gene biology of the genes included in the newly identified loci.

Lead SNP	Genes in the locus	Gene function (GeneCards/Entrez Gene/Uniprot)	Gene expression in kidney (human protein atlas)	OMIM disease (#)	SNP in NHGRI Catalog	PubMed ("gene AND kidney")
rs10874312	intergenic, nearest gene (450kbp) *ADGRL2 (alias LPHN2)*	*ADGRL2* encodes a member of the latrophilin subfamily of G-protein coupled receptors. The encoded protein participates in the regulation of exocytosis.	Low in glomeruli, not detected in tubules	-		-
rs12144044	*RHOC*	This gene encodes a member of the Rho family of small GTPases. Regulates a signal transduction pathway linking plasma membrane receptors to the assembly of focal adhesions and actin stress fibers.	Low in tubules, not detected in glomeruli	-	-	Hutchison et al.[58]: Rho isoforms have distinct and specific functions in the process of epithelial to mesenchymal transition in renal proximal tubular cells.Koshikawa et al. [59] Fasudil, a Rho-kinase inhibitor, reverses L-NAME exacerbated severe nephrosclerosis in spontaneously hypertensive rats.
rs187355703	*LOC100129455*	LOC100129455 is an RNA Gene.	-	-	-	-
rs111366116	*MIR581*	MIR581 (MicroRNA 581) is an RNA Gene, and is affiliated with the miRNA class.	-	-	-	-
rs113246091	*PIK3R1*	Phosphatidylinositol 3-kinase phosphorylates the inositol ring of phosphatidylinositol at the 3-prime position. These products are second messengers in growth signaling pathways. Necessary for the insulin-stimulated increase in glucose uptake and glycogen synthesis in insulin-sensitive tissues.	Medium staining in both glomeruli and tubules	agammaglobulin-emia-7 (MIM #615214), SHORT syndrome (MIM #269880), and immunodeficiency 36 (MIM #16005)	-	Case series report of SHORT Syndrome with ectopic kidney[60]
rs7764488	*EYA4*	Encodes eyes absent homolog 4 protein that may act as a transcriptional activator and has tyrosine phosphatase activity. Roles in eye development have been described. The encoded protein is also a putative oncogene that mediates DNA repair, apoptosis, and innate immunity following DNA damage, cellular damage, and viral attack.	Medium staining in both glomeruli and tubules (differences across antibodies)	deafness, autosomal-dominant 10 (MIM #601316); cardiomyopathy, dilated, 1J (MIM #605362)	-	-
rs13298297	*ASTN2*	This gene encodes a protein that is expressed in the brain and may function in neuronal migration, based on functional studies of the related astrotactin 1 gene in human and mouse. A deletion at this locus has been associated with schizophrenia.	Medium staining in both glomeruli and tubules	-	-	-
	ASTN2-AS1	ASTN2-AS1 (ASTN2 Antisense RNA 1) is an RNA Gene, and is affiliated with the non-coding RNA class.	-	-	-	-

rs1111571	*SLC7A6*	Involved in uptake of dibasic amino acids and some neutral amino acids. Requires coexpression with SLC3A2/4F2hc for uptake of arginine, leucine and glutamine.	Not detected	-	-	Locus identified previously by the CKDGen Consortium in a pathway-based approach[61].
	SLC7A6OS	Encodes Solute Carrier Family 7 Member 6 Opposite Strand. Directs RNA polymerase II nuclear import.	Medium staining in both glomeruli and tubules	=	=	=
	PRMT7	Encodes for protein arginine methyltransferase 7, which catalyzes protein arginine methylation, an irreversible protein modification. Synthesized SDMA. Arginine methylation is implicated in signal transduction, RNA transport, and RNA splicing.	Medium staining in glomeruli, high in tubules	-	-	In mice, susceptibility alleles for doxorubicin nephropathy are associated with reduced prmt7 expression[62].
	PLA2G15	Lysosomal enzyme that has both calcium-independent phospholipase A2 and transacylase activities.	Medium to high staining in glomeruli, high in tubules	-	-	-
	SMPD3	Catalyzes the hydrolysis of sphingomyelin to form ceramide and phosphocholine. Probably participates in bone and dentin mineralization.	Low staining in glomeruli, high in tubules	-	-	-
rs9962915	*EPB41L3*	Tumor suppressor that inhibits cell proliferation and promotes apoptosis. Modulates the activity of protein arginine N-methyltransferases, including PRMT3 and PRMT5.	Medium staining in tubules, not detected in glomeruli	-	-	Differential splicing connected to diverse roles in kidney and brain physiology, and potentially unique functions in cell proliferation and tumor suppression[63].
rs12458009	*CDH20*	Encodes for a calcium dependent cell-cell adhesion glycoprotein.	Medium staining in tubules, not detected in glomeruli	-	-	-

Supplementary Table 7. 1000 Genomes lead variants confirming the 39 known loci. Shown are the variant with the smallest p-value in the 1000 Genomes meta-analysis that reside in previously reported loci[61,64-67].

1000 Genomes lead variant	Chr	Position (bp)	Index Gene	Effect allele	IQ	Effect	SE	P-value	r^2	previously reported lead variant
rs7546668	1	15,855,123	*CASP9*	C	0.99	-0.0063	0.0010	1.14×10^{-9}	1.00	rs12124078
rs10127790	1	109,891,133	*SYPL2*	T	0.99	0.0061	0.0010	7.58×10^{-9}	0.79	rs12136063
rs267738	1	150,940,625	*ANXA9*	T	1.00	-0.0091	0.0011	1.48×10^{-14}	1.00	rs267734
rs3850625	1	201,016,296	*CACNA1S*	A	1.00	0.0088	0.0015	2.24×10^{-8}	Identical	rs3850625
rs807601	2	15,793,014	*DDX1*	T	0.97	0.0067	0.0010	3.84×10^{-11}	0.07	rs6431731
rs780093	2	27,742,603	*GCKR*	T	1.00	0.0081	0.0009	1.57×10^{-16}	0.90	rs1260326
rs4500972	2	73,767,897	*NAT8*	A	0.90	0.0108	0.0012	3.20×10^{-18}	0.89	rs13538
rs1047891*	2	211,540,507	*CPS1*	A	0.90	-0.0089	0.0010	1.90×10^{-16}	Identical	rs7422339
rs7640665	3	141,813,172	*TFDP2*	A	0.93	-0.0072	0.0010	4.66×10^{-11}	0.92	rs347685
rs6809651	3	185,814,642	*ETV5*	A	1.00	-0.0081	0.0014	2.34×10^{-8}	1.00	rs10513801
rs13146355	4	77,412,140	*SHROOM3*	A	1.00	-0.0121	0.0009	3.18×10^{-37}	0.85	rs17319721
rs700236	5	39,367,739	*DAB2*	A	0.99	0.0084	0.0009	1.74×10^{-18}	0.83	rs11959928
rs3812036	5	176,813,404	*SLC34A1*	T	0.93	-0.0102	0.0011	8.90×10^{-19}	0.63	rs6420094
rs1317983	6	43,806,335	*VEGFA*	T	0.92	0.0080	0.0010	1.10×10^{-13}	0.83	rs881858
rs2279463	6	160,668,389	*SLC22A2*	A	0.99	0.0118	0.0014	1.07×10^{-15}	Identical	rs2279463
rs62435145	7	1,286,567	*UNCX*	T	0.60	-0.0077	0.0013	2.71×10^{-8}	0.86	rs10277115
rs112029703	7	77,238,678	*TMEM60*	A	0.98	-0.0065	0.0010	1.38×10^{-9}	0.46	rs6465825
rs10254101	7	151,415,536	*PRKAG2*	T	0.92	-0.0104	0.0011	6.09×10^{-20}	0.96	rs7805747
rs36071802	8	23,715,871	*STC1*	T	0.95	0.0079	0.0009	1.16×10^{-15}	0.73	rs10109414
rs10746942	9	71,434,465	*PIP5K1B*	A	1.00	0.0086	0.0009	3.56×10^{-18}	0.81	rs4744712
rs80282103	10	899,071	*WDR37*	A	0.93	0.0123	0.0017	1.12×10^{-11}	0.62	rs10794720
rs10994856	10	52,645,248	*A1CF*	A	0.97	0.0075	0.0012	4.77×10^{-9}	1.00	rs10994860
rs84178	11	2,774,374	*KCNQ1*	A	0.98	-0.0078	0.0012	4.29×10^{-9}	0.73	rs163160
rs3925584	11	30,760,335	*MPPED2*	T	0.99	-0.0079	0.0009	2.09×10^{-16}	Identical	rs3925584
rs11604462	11	65,551,648	*RNASEH2C*	A	0.99	-0.0060	0.0009	1.90×10^{-9}	1.00	rs4014195
rs11062167	12	364,739	*SLC6A13*	A	0.97	-0.0055	0.0009	1.12×10^{-8}	0.57	rs10774021
rs67551338	12	3,393,100	*TSPAN9*	T	0.91	-0.0124	0.0020	2.17×10^{-9}	0.46	rs10491967
rs9529913	13	72,345,089	*DACH1*	T	0.98	-0.0066	0.0009	2.51×10^{-11}	0.93	rs626277
rs2453533	15	45,641,225	*GATM*	A	1.00	-0.0135	0.0009	2.65×10^{-43}	Identical	rs2453533
15:53922280	15	53,922,280	*WDR72*	A	0.99	0.0083	0.0012	7.20×10^{-11}	0.81	rs491567
rs10851885	15	76,304,503	*UBE2Q2*	A	1.00	0.0081	0.0011	2.92×10^{-12}	0.48	rs1394125
rs77924615	16	20,392,332	*UMOD*	A	0.85	0.0176	0.0013	4.57×10^{-40}	0.27	rs12917707
rs894680	17	19,440,538	*SLC47A1*	A	0.82	-0.0074	0.0010	5.46×10^{-12}	0.97	rs2453580
rs12451586	17	37,633,835	*CDK12*	A	0.83	-0.0092	0.0011	2.78×10^{-15}	0.86	rs11078903
rs9895661	17	59,456,589	*BCAS3*	T	0.92	0.0125	0.0012	4.37×10^{-21}	Identical	rs9895661
rs71359461	18	77,156,103	*NFATC1*	C	0.79	-0.0086	0.0013	3.67×10^{-10}	0.34	rs8091180
rs7247977	19	33,358,355	*SLC7A9*	T	0.98	-0.0070	0.0009	2.35×10^{-12}	0.93	rs12460876
rs6058093	20	33,213,196	*TP53INP2*	A	0.91	-0.0074	0.0010	2.26×10^{-13}	0.69	rs6088580
rs6127099	20	52,731,402	*BCAS1*	A	0.87	-0.0095	0.0011	2.91×10^{-17}	0.46	rs17216707

Positions are given on GRCh build 37. The gene closest to the variant is listed (index gene). IQ is the imputation quality metric computed as median of info score (ImputeV2) or RSQ (minimac) across studies. The effects are given on ln eGFRcrea. The correlation r^2 was computed using the SNAP software available at http://www.broadinstitute.org/mpg/snap/ldsearch.php or as Spearman correlation coefficient in the KORA-F4 study for variants not present in the SNAP database. The effects are given in terms of log(eGFRcrea). * The SNP rs7422339 has merged into rs1047891.

Supplementary Table 8. Summary statistics of the 14 previously published loci that were not genome-wide significant in the 1000 Genomes meta-analysis. Shown are the results of the 14 previously published lead variants[61,64-67] in the 1000 Genomes meta-analysis in up to n=110,517 individuals. Also given is the best 1000 Genomes variant in the respective locus (i.e. 1000 Genomes variant with smallest P-value in the region ± 1 Mb around the published variant) and its correlation to the published variant.

Previously published variant	Chr	Position (bp)	Index Gene	Effect allele	IQ	Effect	SE	P-value	1000 G Variant with lowest p-value	r^2	P-value
rs2802729	1	243,501,763	*SDCCAG8*	A	0.90	-0.0037	0.0009	$2.62x10^{-4}$	rs2783971	0.83	$1.20x10^{-4}$
rs4667594	2	170,008,506	*LRP2*	A	0.99	-0.0033	0.0009	$5.53x10^{-4}$	rs35472707	0.04	$3.93x10^{-6}$
rs2712184	2	217,682,779	*IGFBP2*	A	1.00	-0.0042	0.0009	$1.94x10^{-5}$	rs2541381	0.88	$1.77x10^{-6}$
rs6795744	3	13,906,850	*WNT7A*	A	0.96	0.0045	0.0012	$1.21x10^{-3}$	3:13918234*	0.78	$1.50x10^{-5}$
rs9682041	3	170,091,902	*SKIL*	T	1.00	-0.0033	0.0013	$2.22x10^{-2}$	rs6770214	0.00	$6.23x10^{-4}$
rs228611	4	103,561,709	*MANBA*	A	0.99	-0.0040	0.0009	$4.40x10^{-5}$	4:103573122*	0.90	$9.54x10^{-6}$
rs7759001	6	27,341,409	*ZNF204*	A	0.99	-0.0047	0.0010	$3.49x10^{-5}$	rs9348765	0.74	$1.06x10^{-5}$
rs3750082	7	32,919,927	*AVL9*	A	0.95	0.0027	0.0009	$8.40x10^{-3}$	7:33113699*	0.04	$2.63x10^{-4}$
rs6459680	7	156,258,568	*AC005534.6*	T	0.99	-0.0043	0.0010	$1.36x10^{-4}$	rs6971211	0.00	$6.69x10^{-8}$
rs7956634	12	15,321,194	*RERG*	T	1.00	-0.0059	0.0011	$1.17x10^{-6}$	rs12826808	1.00	$1.36x10^{-7}$
rs1106766	12	57,809,456	*R3HDM2*	T	1.00	0.0060	0.0011	$5.22x10^{-7}$	rs3741414	0.84	$1.59x10^{-7}$
rs2928148	15	41,401,550	*INO80*	A	1.00	0.0039	0.0009	$4.57x10^{-5}$	rs6492982	0.73	$2.39x10^{-6}$
rs164748	16	89,708,292	*DPEP1*	C	0.97	0.0047	0.0009	$2.03x10^{-6}$	rs428232	0.83	$2.87x10^{-7}$
rs11666497	19	38,464,262	*SIPA1L3*	T	0.98	-0.0052	0.0012	$4.16x10^{-5}$	rs151087334	0.04	$2.26x10^{-6}$

Positions are given on GRCh build 37. The gene closest to the variant is listed (index gene). IQ is the imputation quality metric computed as median of info score (ImputeV2) or RSQ (minimac) across studies. The effects are given on log(eGFRcrea). The correlation r^2 is computed by SNAP http://www.broadinstitute.org/mpg/snap/ldsearch.php if available, otherwise as Spearman correlation coefficient computed in the KORA-F4 study. The effects are given on ln eGFRcrea.
*Variants 3:13918234, 4:103573122 and 7:33113699 are INDELs.

Supplementary Table 9. The four lead variants in novel loci identified by the 1000 Genome meta-analysis that were also available in the HapMap panel. Shown are the results of the 1000 Genomes meta-analysis based on up to 110,517 subjects and the results in previous HapMap based analysis on up to 133,806 subjects[65]. Power computations to be compared between the two meta-analyses were based on a true effect size assumed to be the average between the two effect estimates (-0.0056, -0.0049, 0.0055, -0.0057, respectively), a true EAF assumed to be the average between the two EAF estimates (0.27, 0.67, 0.71, or 0.78, respectively), a $5x10^{-8}$ significance level, and the sample size of 110,517 or 133,806, respectively. Effective power was computed based on the effective sample size (sample size multiplied with the imputation quality).

		1000 Genomes meta-analysis						HapMap meta-analysis				
**1000 G lead variant**	**Index Gene**	**P-value**	**Effect**	**98.5% CI**	**IQ**	**I^2**	**Power (effective)**	**P-value**	**Effect**	**IQ**	**I^2**	**Power (effective)**
rs12144044	*RHOC*	$2.87x10^{-8}$	-0.0061	-0.0086; -0.0036	0.96	0	0.67 (0.63)	$6.62 x10^{-7}$	-0.0051	0.86	0	0.85 (0.71)
rs10874312	*LPHN2*	$2.20x10^{-8}$	-0.0057	-0.0082; -0.0032	1.00	19	0.47 (0.47)	$5.60x10^{-6}$	-0.0041	1.00	0	0.72 (0.72)
rs1111571	*SLC7A6*	$6.20x10^{-9}$	0.0061	0.0036; 0.0086	1.00	0	0.67 (0.67)	$1.36 x10^{-7}$	0.0049	1.00	0	0.86 (0.86)
rs12458009	*RNF152*	$2.90x10^{-8}$	-0.0064	-0.0092; -0.0037	1.00	22	0.53 (0.53)	$1.56 x10^{-6}$	-0.0050	1.00	8	0.76 (0.76)

IQ is the imputation quality metric computed as median of info score (ImputeV2) or RSQ (minimac) across studies. EAF is the effect allele frequency. The effects are given on ln eGFRcrea. The 98.5% confidence interval (CI) corresponds to the 95% CI accounting for four independent tests and it is computed as effect ± 2.5 * SE. The gene closest to the variant is listed (index gene). Imputation quality is computed as median of info score (ImputeV2) or RSQ (minimac) across studies. I^2 is the heterogeneity statistic as reported by the meta-analysis software METAL[68].

Supplementary Table 10. Novel reconstituted gene sets from the DEPICT pathway analysis. Shown are the 23 novel reconstituted gene sets with FDR<0.05, which contain at least one of the 10 novel index genes. The reconstituted gene sets are ordered according to their membership in a meta gene set. The column p-value represents the association p-value of the enrichment analysis. Novel index genes in each reconstituted gene set are highlighted in bold.

Reconstituted gene set ID	Reconstituted gene set name	Part of meta gene set	P-value	Reconstituted gene set genes
MP:0002891	increased insulin sensitivity	abnormal glucose homeostasis	3.88×10^{-4}	CELA2B, ENSG00000231013, SCTR, PCK1, SLC16A10, **RNF152**, SLC2A4RG, ATXN7L2, RAPSN, TRIB1
MP:0002078	abnormal glucose homeostasis	abnormal glucose homeostasis	9.01×10^{-4}	PCK1, SCTR, CELA2B, ENSG00000231013, GPT, SLC16A10, FASN, **PIK3R1**, ART3, ACSS2
KEGG_TYPE_II_ DIABETES_MELLITUS	KEGG_TYPE_II_DIABETES_ MELLITUS	abnormal glucose homeostasis	1.11×10^{-3}	ENSG00000231013, CELA2B, SPI1, STC1, SCTR, ZNF793, VEGFA, **ARL15**, KLHL26, FASN
GO:0045834	positive regulation of lipid metabolic process	abnormal glucose homeostasis	1.31×10^{-3}	ODF3L1, WDR72, ENSG00000235488, **RNF152**, ZNF571, FAM53B, PTPN3, ZNF570, C13orf30, EXD1
MP:0010454	abnormal truncus arteriosus septation	cardiac septum development	1.15×10^{-3}	RARB, IGF2, **EYA4**, CYP26A1, BMP4, ACVR2B, LAMA5, DAB2, ZNF420, GLI2
MP:0005627	increased circulating potassium level	decreased urine osmolality	3.84×10^{-4}	TFCP2L1, UMOD, HOXD10, HOXD9, **HOXD8**, SALL1, SLC34A1, KNG1, MAMSTR, LRP2
MP:0002843	decreased systemic arterial blood pressure	decreased urine osmolality	5.54×10^{-4}	UMOD, SLC34A1, PCK1, SLC22A2, HOXD9, DPEP1, KNG1, KCNQ1, ENSG00000175892, **HOXD8**
GO:0006109	regulation of carbohydrate metabolic process	glucan metabolic process	1.08×10^{-3}	ZNF527, **PIK3R1**, HKR1, TP53INP2, FAM47E, ZNF420, ENSG00000223561, CELA2B, ZNF383, XPNPEP3
MP:0000554	abnormal carpal bone morphology	MEIS1 PPI	2.00×10^{-4}	HOXD10, HOXD9, SALL1, DACH1, HOXD4, BMP4, **EYA4**, ADAMTS5, GRB10, KIAA0087
ENSG00000214528	ENSG00000214528 PPI subnetwork	MEIS1 PPI	8.33×10^{-4}	HOXD10, HOXD9, HOXD3, **HOXD8**, ENSG00000237380, ENSG00000175892, HOXD4, HOXD1, ENSG00000224189, SALL1
ENSG00000143995	MEIS1 PPI subnetwork	MEIS1 PPI	1.16×10^{-3}	HOXD10, HOXD9, **HOXD8**, HOXD3, ENSG00000175892, ENSG00000237380, HOXD4, HOXD1, ENSG00000224189, ENSG00000226363

ENSG00000196498	NCOR2 PPI subnetwork	NCOR2 PPI	$5.25{\times}10^{-4}$	JARID2, TRIB1, SNX33, RPRD2, **EYA4**, MAMSTR, **PIK3R1**, NFE2L2, WHAMM, PBRM1
ENSG00000084676	NCOA1 PPI subnetwork	NCOR2 PPI	$5.98{\times}10^{-4}$	CDK12, TRIB1, MED1, A1CF, RAI1, ARNT, FBXL20, TP53INP2, **PIK3R1**, CASZ1
ENSG00000215320	ENSG00000215320 PPI subnetwork	NCOR2 PPI	$1.25{\times}10^{-3}$	NCOA6, KDM5A, JARID2, PARP10, RPRD2, **PIK3R1**, SIPA1L3, R3HDM2, **EYA4**, GGT7
KEGG_PROSTATE_ CANCER	KEGG_PROSTATE_CANCER	transcription regulatory region DNA binding	$1.36{\times}10^{-4}$	PTPN9, HOOK3, CELA2B, PBRM1, RBM47, GRHL2, LARP4B, ADAMTS5, **RHOC**, ENSG00000231013
GO:0009968	negative regulation of signal transduction	transcription regulatory region DNA binding	$1.40{\times}10^{-3}$	ETV5, PITPNC1, FOXH1, PTPN9, R3HDM2, **PIK3R1**, CYP26A1, **ARL15**, MPPED2, SOX21-AS1
GO:0048732	gland development	ureteric bud morphogenesis	$5.59{\times}10^{-4}$	FAM160A1, HOXD10, RERG, GRHL2, ENSG00000203392, SHH, SHROOM3, **HOXD8**, **EYA4**, HOXD9
MP:0002989	small kidney	ureteric bud morphogenesis	$8.74{\times}10^{-4}$	HOXD9, HOXD10, BMP4, **EYA4**, IGF2-AS, DACH1, HOXD4, HOXD3, RARB, LRP2
GO:0001658	branching involved in ureteric bud morphogenesis	ureteric bud morphogenesis	$9.80{\times}10^{-4}$	HOXD10, HOXD9, HOXD4, IGF2-AS, SALL1, HOXD3, ENSG00000203392, HOXD1, SHH, **HOXD8**
MP:0000026	abnormal inner ear morphology	ureteric bud morphogenesis	$1.06{\times}10^{-3}$	DNASE1, SALL1, SOX21-AS1, **EYA4**, KLHDC7A, BMP4, FUT2, SETBP1, CASZ1, SMPD3
GO:0060675	ureteric bud morphogenesis	ureteric bud morphogenesis	$1.09{\times}10^{-3}$	HOXD10, HOXD9, IGF2-AS, HOXD4, HOXD3, DACH1, SALL1, **HOXD8**, HOXD1, SHH
GO:0072006	nephron development	ureteric bud morphogenesis	$1.45{\times}10^{-3}$	ENSG00000229589, DACH1, IGF2-AS, SOX21-AS1, EPS15P1, HOXD9, HOXD4, **EYA4**, DNASE1, ADAMTS5
MP:0000830	abnormal diencephalon morphology	WNT3 PPI	$5.05{\times}10^{-4}$	SALL1, FGFBP3, DACH1, ENSG00000256577, ENSG00000229191, PHTF1, CRLF1, ATP1B3, CYP26A1, **EPB41L3**

Supplementary Table 11. Summary of the 8 independent association signals suggested by joint conditional analysis on reported variants with GCTA. Joint conditional analysis was performed on the 53 previously reported and the 10 novel loci associated with eGFRcrea in the 1000 Genomes meta-analysis on up to 110,517 subjects. Shown are the previously reported variants used for the conditional analysis and the association results of the independent association signals with genome-wide significant variants in the 8 genetic regions after adjustment for the previously reported variants (**Supplementary Table 12**).

			**Novel independent genetic variant**								
								**Results of meta-analysis of novel independent signal**		**Results after conditioning on reported variant**	
**Panel in Supple-mentary Figure 2**	**Previously reported variant**	**EAF known variant**	**Index gene**	**Independent association signals**	**Chr**	**Position (bp)**	**Effect allele (EAF)**	**Effect(SE)**	**P-value**	**Effect(SE)**	**P-value**
A1 & A2	rs6431731	0.05	*DDX1*	rs807601	2	15,792,518	T(0.34)	0.007(0.001)	$3.84x10^{-11}$	0.007(0.001)	$2.39x10^{-8}$
B1 & B2	rs12917707	0.17	*UMOD*	rs77924615	16	20,392,332	A(0.20)	0.018(0.001)	$4.57x10^{-40}$	0.009(0.001)	$8.45x10^{-14}$
C1 & C2	rs2279463	0.88	*SLC22A2*	rs316020	6	160,669,081	A(0.11)	0.012(0.002)	$6.39x10^{-15}$	0.011(0.002)	$1.18x10^{-11}$
D1 & D2	rs2453533	0.39	*GATM*	rs146625690	15	45,623,800	A(0.02)	-0.032(0.004)	$2.27x10^{-12}$	-0.026(0.005)	$1.92x10^{-8}$
E1 & E2	rs4744712	0.60	*PIP5K1B*	rs10746875	9	71,156,949	A(0.14)	0.007(0.001)	$4.40x10^{-7}$	0.008(0.001)	$1.29x10^{-8}$
F1 & F2	rs3925584	0.55	*MPPED2*	rs294345	11	30,666,660	T(0.06)	-0.012(0.002)	$4.15x10^{-8}$	-0.014(0.002)	$3.09x10^{-10}$
G1 & G2	rs9895661	0.81	*BCAS3*	rs8080123	17	59,242,914	T(0.22)	0.010(0.001)	$6.19x10^{-17}$	0.010(0.001)	$2.78x10^{-17}$
H1 & H2	rs17319721	0.43	*SHROOM3*	rs62300882	4	77,439,236	C(0.76)	-0.012(0.001)	$3.53x10^{-23}$	-0.006(0.001)	$2.56x10^{-8}$

Position is reported on GRCh build 37. Chr is chromosome. EAF is the effect allele frequency. The effects are given on ln eGFRcrea.

Supplementary Table 12. Characterization of variants with smallest p-value in independent association signals identified by the joint conditional analysis. In each locus with an independent signal near previously reported variants (indicated by index gene), we examined the primary p-value (from the meta-analysis) and the p-value from the joint conditional analysis (with GCTA) and their influence on the variant effect, the allelic correlation, and the inheritance of the previously reported variant with the variant with smallest p-value in potentially independent association signals.

Index Gene	Description of analysis and rationale for confirmation / rejection of a potentially independent association signal
DDX1	In ***DDX1*** the previously reported lead variant rs6431731[66] had median IQ=0.60 and was not genome-wide significant in the HapMap meta-analysis (primary p-value=3.00x10^{-7}). It achieved a higher IQ in The 1000 Genomes imputed data (IQ=0.82) and a higher p-value for association (primary p-value=1.73x10^{-5}). Nevertheless, 69,988 base pairs upstream, the variant rs807601 is genome-wide significant and well-imputed in the HapMap analysis (p-value=6.60x10^{-12}, IQ=0.98) and in the 1000 Genomes meta-analysis (p-value=3.84x10^{-11}, IQ=0.97) and also genome-wide significant after adjusting for the previously reported variant (p-value=2.39x10^{-8}), as well. There is no change of effect size (effect=0.007 in both analyses). The potentially independent association signal and the previously reported variant are uncorrelated (r^2=0.04) but inherit their risk alleles together as the coinheritance indicator D' is high (D'=0.79) (**Supplementary Figure 2 A1-A2**). Thus, compared to previous analysis, the current 1000 Genomes meta-analysis identifies a better index SNP for this locus.
UMOD	In the ***UMOD*** locus the highly significant lead variant rs77924615 (p-value=4.57x10^{-40}) was newly introduced with The 1000 Genomes reference data. The joint conditional analysis suggests that it is independent (conditional p-value=8.45x10^{-14}) from the reported variant rs12917707[64] (p-value=2.01x10^{-34}). Its effect is diminished in the joint conditional analysis (from 0.018 to 0.009). The variants are independent from each other (r^2=0.34) but they are likely to inherit their alleles via the same haplotype given the presence of moderate LD (D'=0.64, **Supplementary Figure 2 B1-B2)**.
SLC22A2	In ***SLC22A2***, rs316020 (primary p-value=6.39x10^{-15}, conditional p-value=1.18x10^{-11}) is suggested to be independent from the previously reported variant rs2279463[67] (primary p-value=1.07x10^{-17}). The effect of rs316020 does not change in, when adjusting on the reported variant (changes from 0.012 to 0.011). The variants have limited correlation but they are in total disequilibrium (r^2=0.45, D'=1.00), suggesting, the risk alleles at the two variants are inherited on the same haplotype (**Supplementary Figure 2 C1-C2).**
GATM	The joint conditional analysis shows that rs146625690 (primary p-value=2.27x10^{-12}) is independent (conditional p-value=1.92x10^{-8}) from the previously reported variant rs2453533[64] (primary p-value=2.65x10^{-43}) in the ***GATM*** locus. The effects of rs146625690 are little diminished when adjusting for the previously reported variant (changes from -0.032 to -0.026). Thus, it is suggested, that rs146625690 is the SNP with lowest p-value in an independent signal. The variants are highly correlated and their risk alleles are inherited via the same haplotype (r^2=0.98, D'=0.85, **Supplementary Figure 2 D1-D2)**.

PIP5K1B	We found that rs10746875 (primary p-value=4.4×10^{-7}) is independent (conditional p-value=1.29×10^{-8}) from the previously reported variant rs4744712[67] (primary p-value=6.93×10^{-18}) in ***PIP5K1B***. Its effect is comparable (changes from 0.007 to 0.008), when adjusting for the previously reported variant in the joint conditional analysis. The variants are uncorrelated (r^2<0.01) and their risk alleles are inherited on different haplotypes (D'=0.12, see **Supplementary Figure 2 E1-E2)**.
MPPED2	The genome-wide significant previously reported variant in the ***MPPED2*** locus is rs3925584[66] (primary p-value=2.09×10^{-16}). The variant rs294345 (primary p-value=4.15×10^{-8}) is independent from the rs3925584 (conditional p-value=3.09×10^{-10}) and its effect is comparable in the joint conditional, when adjusting for the previously reported variant (changes from -0.012 to -0.014). The variant rs294345 is closer to the gene promoter and in the same recombination segment as the previously reported SNP rs3925584. The r^2 and D' between the two variants are low (r^2=0.01, D'=0.42), suggesting independence and that the risk alleles are inherited by separate haplotypes. (**Supplementary Figure 2 F1-F2)**.
BCAS3	The previously reported variant in ***BCAS3*** is rs9895661[67] (primary p-value=4.7×10^{-21}). The lead variant in the independent signal is rs8080123 (primary p-value=6.19×10^{-17}, conditional p-value=2.78×10^{-17}). Its effects are equal in the primary meta-analysis and the joint conditional analyses (both 0.010). The variants are uncorrelated (r^2<0.01), in linkage equilibrium D' (D'=0.09), and are separated by a recombination hotspot (~50 cM/Mb at chr17:59.3MB): for these reasons, their risk alleles are inherited via different haplotypes (**Supplementary Figure 2 G1-G2**).
SHROOM3	Independence from the previously reported variant rs17319721[64] (primary p-value=8.97×10^{-35}) in ***SHROOM3*** is suggested in the joint conditional analysis: the effect of variant of rs62300882 (primary p-value=3.53×10^{-23}, conditional p-value=2.56×10^{-8}) is degraded from -0.012 to -0.006 in the joint conditional analysis. The variants show a low correlation and it is suggested that their risk alleles are inherited via the same haplotype (R^2=0.19, D'=0.90, **Supplementary Figure 2 H1-H2**).

The r^2 and D' are measures for linkage disequilibrium. IQ is the imputation quality metric computed as median of info score (ImputeV2) or RSQ (minimac) across studies. The effects are given on ln eGFRcrea. Recombination rate cM/Mb is given as centimorgan per Megabase.

Supplementary Table 13. Gene biology of the lead variants in the additional signals. The addition signals were identified by the joint conditional analysis using GCTA (**Supplementary Table 11**).

Index SNP	Genes in the locus	Gene function (GeneCards/Entrez Gene/Uniprot)	Gene expression in kidney (human protein atlas)	OMIM disease (#)	SNP in NHGRI Catalog	PubMed ("gene AND kidney")
rs807603	*DDX1*	DEAD (Asp-Glu-Ala-Asp) box helicase 1. Acts as an ATP-dependent RNA helicase, able to unwind both RNA-RNA and RNA-DNA duplexes.	Medium to high staining in both glomeruli and tubules	-	-	Locus has been identified in a GWAS of eGFRcrea in European ancestry participants[66]
rs77924615	*UMOD*	The protein encoded by this gene is the most abundant protein in mammalian urine under physiological conditions. It may act as a constitutive inhibitor of calcium crystallization in renal fluids. Excretion of this protein in urine may provide defense against urinary tract infections caused by uropathogenic bacteria.	High staining in tubules, not detected in glomeruli	Glomerulocystic kidney disease with hyperuricemia and isosthenuria (MIM # 609886), Hyperuricemic nephropathy, familial juvenile 1 (MIM # 162000), Medullary cystic kidney disease 2 (MIM # 603860)	-	Hundreds of entries
	PDILT	Probable redox-inactive chaperone involved in spermatogenesis.	not detected	not found in OMIM	-	Variants in *PDILT* associate with urinary uromodulin levels in GWAS of European ancestry individuals[69]
rs316020	*SLC22A2*	Solute carrier family 22 (organic cation transporter), member 2 is a polyspecific organic cation transporters in the liver, kidney, intestine, and other organs are critical for elimination of many endogenous small organic cations as well as a wide array of drugs and environmental toxins. It is found primarily in the kidney.	High staining in tubules, not detected in glomeruli	-	Associated with blood metabolite ratios[70]	>100 entries, associated with the tubular secretion of creatinine[71]
rs146625690	*GATM*	This gene encodes a mitochondrial enzyme that belongs to the amidinotransferase family (L-arginine:glycine amidinotransferase). This enzyme is involved in creatine biosynthesis, whereby it catalyzes the transfer of a guanido group from L-arginine to glycine, resulting in guanidinoacetic acid, the immediate precursor of creatine.	High staining in tubules, not detected in glomeruli	Cerebral creatine deficiency syndrome 3 (MIM #612718)	-	The locus has been identified in multiple GWAS of eGFR and serum creatinine[64,72,73]
rs10746875	*PIP5K1B*	Encodes phosphatidylinositol-4-phosphate 5-kinase, type I, beta. Participates in the biosynthesis of phosphatidylinositol 4,5-bisphosphate.	Medium staining in tubules, not detected in glomeruli	=	-	The locus has been identified in numerous GWAS of kidney function[67,73,74]
	TMEM252	transmembrane protein 252., protein coding gene	Medium staining in both glomeruli and tubules	not in OMIM	=	-
rs294345	*MPPED2*	This gene likely encodes a metallophosphoesterase. The encoded protein may play a role a brain development.	Medium staining in tubules, not detected in glomeruli	=	=	The locus has been identified in GWAS of eGFRcrea among European ancestry individuals[66]

	DCDC5	This gene encodes a member of the doublecortin family. The doublecortin domain has been demonstrated to bind tubulin and enhance microtubule polymerization.	Not entry for this gene	-	-	The locus has been identified in a GWAS or serum magnesium levels[75].
rs8080123	*TBX2*	Encodes a T-box binding transcription factor with a role in developmental processes.	High staining in both glomeruli and tubules	-	-	One paper describing a role of TBX2 in defining the territory of the pronephric nephron using Xenopus model organism[76].
	BCAS3	Plays a role in angiogenesis. Participates in the regulation of cell polarity and migration. Functions as a transcriptional coactivator of estrogen receptor-responsive genes. Stimulates histone acetyltransferase activity. Binds to chromatin.	Medium staining in both glomeruli and tubules	-	-	SNPs have been identified in GWAS of renal function in American Indians (albuminuria)[74], African Americans (eGFRcrea)[73] and individuals of European ancestry (eGFRcrea)[67]
rs62300882	*SHROOM3*	This gene encodes a PDZ-domain-containing protein that belongs to a family of Shroom-related proteins. Controls cell shape changes in the neuroepithelium during neural tube closure. Induces apical constriction in epithelial cells by promoting the apical accumulation of F-actin and myosin II, and probably by bundling stress fibers.	Medium staining in tubules, low in glomeruli	-	=	Dysfunction of Shroom3 leads to renal injury in the FHH rat, knockdown causes glomerular defects in zebrafish, and variants in humans associate with impairment of the glomerular filtration barrier[77]. In mice, Shroom3 is required for normal podocyte architecture and function[78] Locus identified in GWAS of serum magnesium levels[75] and eGFRcrea[64]

Supplementary Table 14. Polygenic risk score analysis in the TRAILS study. Shown are the results of the polygenic risk score (PRS) analysis of variants associated with eGFRcrea at a given p-value threshold in the 1000 Genomes meta-analysis. PRS analysis was conducted on 1000 Genomes imputed data of the TRAILS (n=1,071) study: an independent study, which was not part of the meta-analysis. Shown are the numbers of variants, p-value thresholds, observed variance explained, and association p-value of the respective PRS analyses.

P-value threshold	Number of variants	Observed variance explained	PRS association p-value
$P < 5x10^{-8}$	60	0.013	$2.0x10^{-4}$
$P < 5x10^{-7}$	77	0.015	$6.0x10^{-5}$
$P < 5x10^{-6}$	124	0.014	$1.0x10^{-4}$
$P < 5x10^{-5}$	327	0.022	$1.3x10^{-6}$
$P < 5x10^{-4}$	1,312	0.017	$1.8x10^{-5}$
$P < 5x10^{-3}$	7,448	0.010	$8.1x10^{-4}$
P < 0.05	45,949	0.003	$7.6x10^{-2}$
P < 0.5	231,457	0.003	$7.9x10^{-2}$
P < 1	312,083	0.004	$5.3x10^{-2}$

NESDA and TRAILS were imputed to the 1000G reference panels (cosmopolitan panel of phase 1 version 3, release March 2012) using minimac and IMPUTEV2, respectively.

Supplementary Table 15. Expression quantitative trait loci (eQTL) lookup. Shown are potentially functional implications for the significant SNPs or their proxies in the two loci (*SLC7A6* and *RHOC*), for which eQTL associations were revealed.

1000 Genomes lead variant	Chr	Position (bp)	Probe Name	Probe Center Position (bp)	Effect/ Non-effect allele	Effect Direction	Gene Name	P-Value
rs1111571	16	66,920,682	1430347	66,892,846	G/A	-	*SLC7A6, SLC7A6OS*	$1.96x10^{-59}$
rs1111571	16	66,920,682	5900286	67,158,448	G/A	-	*ZFP90*	$3.68x10^{-30}$
rs1111571	16	66,920,682	6380364	66,891,750	G/A	-	*SLC7A6*	$6.73x10^{-26}$
rs1111571	16	66,920,682	6480037	66,852,243	G/A	+	*LYPLA3*	$1.54x10^{-5}$
rs1111571	16	66,920,682	2260255	66,783,041	G/A	-	*NFATC3*	$2.46x10^{-4}$
rs12144044	1	113,050,314	4390619	113,045,326	A/C	-	*RHOC*	$2.60x10^{-15}$
rs12144044	1	113,050,314	4250327	113,045,386	A/C	-	*RHOC*	$1.16x10^{-12}$
rs12144044	1	113,050,314	3610164	112,886,029	A/C	+	*ST7L*	$3.40x10^{-3}$

Chr is chromosome. Position is given on GRCh build 37. P-value is the result of the cis eQTL association whereas the corresponding Effect Direction is based on the Effect Allele.

Study specific acknowledgements and funding sources for participating studies, alphabetical order.

3C. *Three-City Study*. The work was made possible by the participation of the control subjects, the patients, and their families. We thank Dr. Anne Boland (CNG) for her technical help in preparing the DNA samples for analyses. This work was supported by the National Foundation for Alzheimer's disease and related disorders, the Institut Pasteur de Lille and the Centre National de Génotypage. The 3C Study was performed as part of a collaboration between the Institut National de la Santé et de la Recherche Médicale (Inserm), the Victor Segalen Bordeaux II University and Sanofi-Synthélabo. The Fondation pour la Recherche Médicale funded the preparation and initiation of the study. The 3C Study was also funded by the Caisse Nationale Maladie des Travailleurs Salariés, Direction Générale de la Santé, MGEN, Institut de la Longévité, Agence Française de Sécurité Sanitaire des Produits de Santé, the Aquitaine and Bourgogne Regional Councils, Fondation de France and the joint French Ministry of Research/INSERM "Cohortes et collections de données biologiques" programme. Lille Génopôle received an unconditional grant from Eisai.

AGES. *Age, Gene/Environment Susceptibility-Reykjavik Study*. This study has been funded by NIH contract N01-AG-1-2100, the NIA Intramural Research Program, Hjartavernd (the Icelandic Heart Association), and the Althingi (the Icelandic Parliament). The study is approved by the Icelandic National Bioethics Committee, VSN: 00-063. The researchers are indebted to the participants for their willingness to participate in the study.

ARIC. *Atherosclerosis Risk in Communities study*. The ARIC study is carried out as a collaborative study supported by National Heart, Lung, and Blood Institute contracts (HHSN268201100005C, HHSN268201100006C, HHSN268201100007C, HHSN268201100008C, HHSN268201100009C, HHSN268201100010C, HHSN268201100011C, and HHSN268201100012C), R01HL087641, R01HL59367 and R01HL086694; National Human Genome Research Institute contract U01HG004402; and National Institutes of Health contract HHSN268200625226C. The authors thank the staff and participants of the ARIC study for their important contributions. Infrastructure was partly supported by Grant Number UL1RR025005, a component of the National Institutes of Health and NIH Roadmap for Medical Research. This work as well as YL and AK were supported by the German Research Foundation (KO 3598/2-1, KO 3598/3-1 and CRC1140 A05 to AK).

ASPS. *Austrian Stroke Prevention Study*. The research reported in this article was funded by the Austrian Science Fond (FWF) grant number P20545-P05 and P13180. The Medical University of Graz supports the databank of the ASPS. The authors thank the staff and the participants of the ASPS for their valuable contributions. We thank Birgit Reinhart for her long-term administrative commitment and Ing Johann Semmler for the technical assistance at creating the DNA-bank.

BMES. *Blue Mountains Eye Study*. The BMES has been supported by the Australian RADGAC grant (1992-94) and Australian National Health & Medical Research Council, Canberra Australia (Grant Nos: 974159, 211069, 991407, 457349). The GWAS studies of Blue Mountains Eye Study population are supported by the Australian National Health & Medical Research Council (Grant Nos: 512423, 475604, 529912) and the Wellcome Trust, UK (2008). EGH and JJW are funded by the Australian National Health & Medical Research Council Fellowship Schemes.

CILENTO. *Italian Network on Genetic Isolates – Cilento.* We thank the populations of Cilento for their participation in the study. The study was supported by the Italian Ministry of Universities and CNR

(PON03PE_00060_7, Interomics Flagship Project), the Assessorato Ricerca Regione Campania, the Fondazione con il SUD (2011-PDR-13), and the Istituto Banco di Napoli - Fondazione to MC.

COLAUS. The CoLaus authors thank Yolande Barreau, Mathieu Firmann, Vladimir Mayor, Anne-Lise Bastian, Binasa Ramic, Martine Moranville, Martine Baumer, Marcy Sagette, Jeanne Ecoffey and Sylvie Mermoud for data collection. The CoLaus study received financial contributions from GlaxoSmithKline, the Faculty of Biology and Medicine of Lausanne, the Swiss National Science Foundation (33CSCO-122661, 3200BO-111361/2, 3100AO-116323/1, 310000-112552). The computations for CoLaus imputation were performed in part at the Vital-IT center for high performance computing of the Swiss Institute of Bioinformatics. We thank Vincent Mooser for his contribution to the CoLaus study.

EGCUT. *Estonian Genome Center University of Tartu*. EGCUT received financing from FP7 grants (278913, 306031, 313010) and targeted financing from Estonian Government (SF0180142s08). EGCUT studies were covered from Infra-structure grant no. 3.2.0304.11-0312 funded mostly by the European Regional Development Fund, Center of Excellence in Genomics (EXCEGEN) and University of Tartu (SP1GVARENG). We acknowledge EGCUT technical personnel, especially Mr V. Soo and S. Smit. Data analyses were carried out in part in the High Performance Computing Center of the University of Tartu.

FamHS. *Family Heart Study*. The FHS work was supported in part by NIH grants 5R01HL08770003, 5R01HL08821502 (Michael A. Province) from the NHLBI and 5R01DK07568102, 5R01DK06833603 from the NIDDK (I.B.B.). The authors thank the staff and participants of the FamHS for their important contributions.

FHS. *Framingham Heart Study*. This research was conducted in part using data and resources from the Framingham Heart Study of the National Heart Lung and Blood Institute of the National Institutes of Health and Boston University School of Medicine. The analyses reflect intellectual input and resource development from the Framingham Heart Study investigators participating in the SNP Health Association Resource (SHARe) project. This work was partially supported by the National Heart, Lung and Blood Institute's Framingham Heart Study (Contract No. N01-HC-25195) and its contract with Affymetrix, Inc. for genotyping services (Contract No. N02-HL-6-4278). A portion of this research utilized the Linux Cluster for Genetic Analysis (LinGA-II) funded by the Robert Dawson Evans Endowment of the Department of Medicine at Boston University School of Medicine and Boston Medical Center.

GENDIAN. *GENetics of DIAbetic Nephropathy study*. The support of the physicians, the patients, and the staff of the Diabetes Zentrum Mergentheim (Head: Prof. Dr. Thomas Haak), the diabetes outpatient clinic Dr Nusser - Dr Kreisel, the dialysis centers KfH Amberg, KfH Bayreuth, KfH Deggendorf, KfH Donauwörth, KfH Freising, KfH Freyung, KfH Fürth, KfH Hof, KfH Ingolstadt, KfH Kelheim, KfH München Elsenheimerstraße, KfH München-Schwabing, KfH Neumarkt, KfH Neusäß, KfH Oberschleißheim, KfH Passau, KfH Plauen, KfH Regensburg Günzstraße, KfH Regensburg Caritas-Krankenhaus, KfH Straubing, KfH Sulzbach-Rosenberg, KfH Weiden, Dialysezentrum Augsburg Dr. Kirschner, Dialysezentrum Bad Alexandersbad, KfH Bamberg, Dialysezentrum Emmering, Dialysezentrum Klinikum Landshut, Dialysezentrum Landshut, Dialysezentrum Pfarrkirchen, Dialysezentrum Schwandorf, Dr. Angela Götz, the medical doctoral student Johanna Christ and the Study Nurse Ingrid Lugauer. The expert technical assistance of Claudia Strohmeier is acknowledged. Phenotyping was funded by the Dr. Robert Pfleger-Stiftung (Dr Carsten A. Böger), the MSD Stipend Diabetes (Dr Carsten A. Böger) and the University Hospital of Regensburg (intramural grant ReForM A to Dr. A. Götz, ReForM C to Dr. Carsten Böger). Genome-wide genotyping was funded by the KfH Stiftung Präventivmedizin e.V. (Dr. Carsten A. Böger, Dr. Jens Brüning), the Else Kröner-Fresenius-Stiftung (2012_A147 to Dr Carsten A. Böger and Dr Iris M. Heid) and the University Hospital Regensburg (Dr Carsten A. Böger). Data analysis was funded by the Else

Kröner-Fresenius Stiftung (Dr. Iris M. Heid and Dr. Carsten A. Böger: 2012_A147; Dr. Carsten A. Böger and Dr. Bernhard K. Krämer: P48/08//A11/08). GENDIAN Study Group: Mathias Gorski, Iris M. Heid, Bernhard K. Krämer, Myriam Rheinberger, Michael Broll, Alexander Lammert, Jens Brüning, Matthias Olden, Klaus Stark, Claudia Strohmeier, Simone Neumeier, Sarah Hufnagel, Petra Jackermeier, Emilia Ruff, Johanna Christ, Peter Nürnberg, Thomas Haak, Carsten A. Böger.

HABC. *Health Aging and Body Composition Study*. The HABC study was funded by the National Institutes of Aging. This research was supported by NIA contracts N01AG62101, N01AG62103, and N01AG62106. The genome-wide association study was funded by NIA grant 1R01AG032098-01A1 to Wake Forest University Health Sciences and genotyping services were provided by the Center for Inherited Disease Research (CIDR). CIDR is fully funded through a federal contract from the National Institutes of Health to The Johns Hopkins University, contract number HHSN268200782096C. This research was supported in part by the Intramural Research Program of the NIH, National Institute on Aging.

HCS. *Hunter Community Study*. The University of Newcastle provided $300,000 from its Strategic Initiatives Fund, and $600,000 from the Gladys M Brawn Senior Research Fellowship scheme; Vincent Fairfax Family Foundation, a private philanthropic trust, provided $195,000; The Hunter Medical Research Institute provided media support during the initial recruitment of participants; and Dr Anne Crotty, Prof. Rodney Scott and Associate Prof. Levi provided financial support towards freezing costs for the long-term storage of participant blood samples. The authors would like to thank the men and women participating in the HCS as well as all the staff, investigators and collaborators who have supported or been involved in the project to date. A special thank you should go to Alison Koschel and Debbie Quain who were instrumental in setting up the pilot study and initial phase of the project.

HPFS. *Health Professionals Follow-Up Study.* The NHS/HPFS type 2 diabetes GWAS (U01HG004399) is a component of a collaborative project that includes 13 other GWAS (U01HG004738, U01HG004422, U01HG004402, U01HG004729, U01HG004726, U01HG004735, U01HG004415, U01HG004436, U01HG004423, U01HG004728, RFAHG006033; National Institute of Dental & Craniofacial Research: U01DE018993, U01DE018903) funded as part of the Gene Environment-Association Studies (GENEVA) under the NIH Genes, Environment and Health Initiative (GEI). Assistance with phenotype harmonization and genotype cleaning, as well as with general study coordination, was provided by the GENEVA Coordinating Center (U01HG004446). Assistance with data cleaning was provided by the National Center for Biotechnology Information. Genotyping was performed at the Broad Institute of MIT and Harvard, with funding support from the NIH GEI (U01HG04424), and Johns Hopkins University Center for Inherited Disease Research, with support from the NIH GEI (U01HG004438) and the NIH contract "High throughput genotyping for studying the genetic contributions to human disease"(HHSN268200782096C). Additional funding for the current research was provided by the National Cancer Institute (P01CA087969, P01CA055075), and the National Institute of Diabetes and Digestive and Kidney Diseases (R01DK058845). We thank the staff and participants of the NHS and HPFS for their dedication and commitment.

INGI-CARLANTINO. *Italian Network on Genetic Isolates – Carlantino.* We thank Anna Morgan and Angela D'Eustacchio for technical support. We are grateful to the municipal administrators for their collaboration on the project and for logistic support. We thank all participants to this study.

INGI-FVG. *Italian Network on Genetic Isolates – Friuli Venezia-Giulia.* We thank Anna Morgan and Angela D'Eustacchio for technical support. We are grateful to the municipal administrators for their collaboration on the project and for logistic support. We thank all participants to this study.

INGI-VAL BORBERA. *Italian Network on Genetic Isolates – Val Borbera*. We thank the inhabitants of the Val Borbera who made this study possible, the local administrations and the ASL-Novi Ligure (Al) for support. We also thank Clara Camaschella for data collection supervision and organization of the clinical data collection, Fiammetta Vigano` for technical help and Corrado Masciullo for building the analysis platform. The research was supported by funds from Compagnia di San Paolo, Torino, Italy; Fondazione Cariplo, Italy and Ministry of Health, Ricerca Finalizzata 2008 and 2011/2012, CCM 2010, PRIN 2009 and Telethon, Italy to DT.

IPM. *Mount Sinai BioMe Biobank Program*. The Mount Sinai BioMe Biobank Program is supported by The Andrea and Charles Bronfman Philanthropies.

KORA-F3 and **F4**. The genetic epidemiological work was funded by the NIH subcontract from the Children's Hospital, Boston, US, (H.E.W., I.M.H, prime grant 1 R01 DK075787-01A1), the German National Genome Research Net NGFN2 and NGFNplus (H.E.W. 01GS0823; WK project A3, number 01GS0834), the Munich Center of Health Sciences (MC Health) as part of LMUinnovativ, and by the Else Kröner-Fresenius-Stiftung (P48/08//A11/08; C.A.B., B.K.K; 2012_A147 to CAB and IMH.). The Genetic Epidemiology at the University of Regensburg received financial contributions from the BMBF (01ER1206 and 01ER1507). The kidney parameter measurements in F3 were funded by the Else Kröner-Fresenius-Stiftung (C.A.B., B.K.K.) and the Regensburg University Medical Center, Germany; in F4 by the University of Ulm, Germany (W.K.). Genome wide genotyping costs in F3 and F4 were in part funded by the Else Kröner-Fresenius-Stiftung (C.A.B., B.K.K.). *De novo* genotyping in F3 and F4 were funded by the Else Kröner-Fresenius-Stiftung (C.A.B., B.K.K.). The KORA research platform and the MONICA Augsburg studies were initiated and financed by the Helmholtz Zentrum München, German Research Center for Environmental Health, by the German Federal Ministry of Education and Research and by the State of Bavaria. Genotyping was performed in the Genome Analysis Center (GAC) of the Helmholtz Zentrum München. The LINUX platform for computation were funded by the University of Regensburg for the Department of Epidemiology and Preventive Medicine at the Regensburg University Medical Center.

LIFELINES. The authors wish to acknowledge the services of the Lifelines Cohort Study, the contributing research centers delivering data to Lifelines, and all the study participants. Lifelines group authors: Behrooz Z Alizadeh[1], H Marike Boezen[1], Lude Franke[2], Pim van der Harst[3], Gerjan Navis[4], Marianne Rots[5], Harold Snieder[1], Morris Swertz[2], Bruce HR Wolffenbuttel[6] and Cisca Wijmenga[2]

1. Department of Epidemiology, University of Groningen, University Medical Center Groningen, The Netherlands
2. Department of Genetics, University of Groningen, University Medical Center Groningen, The Netherlands
3. Department of Cardiology, University of Groningen, University Medical Center Groningen, The Netherlands
4. Department of Internal Medicine, Division of Nephrology, University of Groningen, University Medical Center Groningen, The Netherlands
5. Department of Medical Biology, University of Groningen, University Medical Center Groningen, The Netherlands
6. Department of Endocrinology, University of Groningen, University Medical Center Groningen, The Netherlands

MESA. *Multi-Ethnic Study of Atherosclerosis*. University of Washington (N01-HC-95159),Regents of the University of California (N01-HC-95160), Columbia University (N01-HC-95161), Johns Hopkins University

(N01-HC-95162, N01-HC-95168), University of Minnesota (N01-HC-95163), Northwestern University (N01-HC-95164), Wake Forest University (N01-HC-95165), University of Vermont (N01-HC-95166), New England Medical Center (N01-HC-95167), Harbor-UCLA Research and Education Institute (N01-HC-95169), Cedars-Sinai Medical Center (R01-HL-071205), University of Virginia (subcontract to R01-HL-071205)

MICROS. *Microisolates in South Tyrol study*. We owe a debt of gratitude to all participants. We thank the primary care practitioners R. Stocker, S. Waldner, T. Pizzecco, J. Plangger, U. Marcadent and the personnel of the Hospital of Silandro (Department of Laboratory Medicine) for their participation and collaboration in the research project. In South Tyrol, the study was supported by the Ministry of Health and Department of Educational Assistance, University and Research of the Autonomous Province of Bolzano, the South Tyrolean Sparkasse Foundation, and the European Union framework program 6 EUROSPAN project (contract no. LSHG-CT-2006-018947).

NESDA. *The Netherlands Study of Depression and Anxiety*. The infrastructure for the NESDA study is funded through the Geestkracht programme of the Dutch Scientific Organization (ZON-MW, grant number 10-000-1002) and matching funds from participating universities and mental health care organizations. Genotyping in NESDA was funded by the Genetic Association Information Network (GAIN) of the Foundation for the US National Institutes of Health.

NHS. *Nurses' Health Study*. The NHS/HPFS type 2 diabetes GWAS (U01HG004399) is a component of a collaborative project that includes 13 other GWAS (U01HG004738, U01HG004422, U01HG004402, U01HG004729, U01HG004726, U01HG004735, U01HG004415, U01HG004436, U01HG004423, U01HG004728, RFAHG006033; National Institute of Dental & Craniofacial Research: U01DE018993, U01DE018903) funded as part of the Gene Environment-Association Studies (GENEVA) under the NIH Genes, Environment and Health Initiative (GEI). Assistance with phenotype harmonization and genotype cleaning, as well as with general study coordination, was provided by the GENEVA Coordinating Center (U01HG004446). Assistance with data cleaning was provided by the National Center for Biotechnology Information. Genotyping was performed at the Broad Institute of MIT and Harvard, with funding support from the NIH GEI (U01HG04424), and Johns Hopkins University Center for Inherited Disease Research, with support from the NIH GEI (U01HG004438) and the NIH contract "High throughput genotyping for studying the genetic contributions to human disease”(HHSN268200782096C). The NHS renal function and albuminuria work was supported by DK66574. Additional funding for the current research was provided by the National Cancer Institute (P01CA087969, P01CA055075), and the National Institute of Diabetes and Digestive and Kidney Diseases (R01DK058845). We thank the staff and participants of the NHS and HPFS for their dedication and commitment.

NSPHS. *The Northern Swedish Population Health Study*. The NSPHS was supported by grants from the Swedish Natural Sciences Research Council, the European Union through the EUROSPAN project (contract no. LSHG-CT-2006-018947), the Foundation for Strategic Research (SSF) and the Linneaus Centre for Bioinformatics (LCB). We are also grateful for the contribution of samples from the Medical Biobank in Umeå and for the contribution of the district nurse Svea Hennix in the Karesuando study.

RS-I. *The Rotterdam Study*. The GWA study was funded by the Netherlands Organisation of Scientific Research NWO Investments (nr. 175.010.2005.011, 911-03-012), the Research Institute for Diseases in the Elderly (014-93-015; RIDE2), the Netherlands Genomics Initiative (NGI)/Netherlands Consortium for Healthy Aging (NCHA) project nr. 050-060-810. We thank Pascal Arp, Mila Jhamai, Dr Michael

Moorhouse, Marijn Verkerk, and Sander Bervoets for their help in creating the GWAS database. The Rotterdam Study is funded by Erasmus Medical Center and Erasmus University, Rotterdam, Netherlands Organization for the Health Research and Development (ZonMw), the Research Institute for Diseases in the Elderly (RIDE), the Ministry of Education, Culture and Science, the Ministry for Health, Welfare and Sports, the European Commission (DG XII), and the Municipality of Rotterdam. The authors are very grateful to the participants and staff from the Rotterdam Study, the participating general practitioners and the pharmacists. We would like to thank Dr. Tobias A. Knoch, Luc V. de Zeeuw, Anis Abuseiris, and Rob de Graaf as well as their institutions the Erasmus Computing Grid, Rotterdam, The Netherlands, and especially the national German MediGRID and Services@MediGRID part of the German D-Grid, both funded by the German Bundesministerium fuer Forschung und Technology under grants #01 AK 803 A-H and # 01 IG 07015 G, for access to their grid resources. Abbas Dehghan is supported by NWO grant (vici, 918-76-619).

SAPALDIA. *Swiss Study on Air Pollution and Lung Diseases in Adults*. The SAPALDIA Team: Study directorate: T Rochat (p), NM Probst Hensch (e/g), N Künzli (e/exp), C Schindler (s), JM Gaspoz (c) Scientific team: JC Barthélémy (c), W Berger (g), R Bettschart (p), A Bircher (a), O Brändli (p), C Brombach (n), M Brutsche (p), L Burdet (p), M Frey (p), U Frey (pd), MW Gerbase (p), D Gold (e/c/p), E de Groot (c), W Karrer (p), R Keller (p), B Martin (pa), D Miedinger (o), U Neu (exp), L Nicod (p), M Pons (p), F Roche (c), T Rothe (p), E Russi (p), P Schmid-Grendelmeyer (a), A Schmidt-Trucksäss (pa), A Turk (p), J Schwartz (e), D. Stolz (p), P Straehl (exp), JM Tschopp (p), A von Eckardstein (cc), E Zemp Stutz (e). Scientific team at coordinating centers: M Adam (e/g), C Autenrieth (pa), PO Bridevaux (p), D Carballo (c), E Corradi (exp), I Curjuric (e), J Dratva (e), A Di Pasquale (s), E Dupuis Lozeron (s), E Fischer (e), M Germond (s), L Grize (s), D Keidel (s), S Kriemler (pa), A Kumar (g), M Imboden (g), N Maire (s), A Mehta (e), H Phuleria (exp), E Schaffner (s), GA Thun (g) A Ineichen (exp), M Ragettli (e), M Ritter (exp), T Schikowski (e), M Tarantino (s), M Tsai (exp) (a) allergology, (c) cardiology, (cc) clinical chemistry, (e) epidemiology, (exp) exposure, (g) genetic and molecular biology, (m) meteorology, (n) nutrition, (o) occupational health, (p) pneumology, (pa) physical activity, (pd) pediatrics, (s) statistics. Funding: The Swiss National Science Foundation (grants no 33CSCO-134276/1, 33CSCO-108796, 3247BO-104283, 3247BO-104288, 3247BO-104284, 3247-065896, 3100-059302, 3200-052720, 3200-042532, 4026-028099), the Federal Office for Forest, Environment and Landscape, the Federal Office of Public Health, the Federal Office of Roads and Transport, the canton's government of Aargau, Basel-Stadt, Basel-Land, Geneva, Luzern, Ticino, Valais, and Zürich, the Swiss Lung League, the canton's Lung League of Basel Stadt/ Basel Landschaft, Geneva, Ticino, Valais and Zurich, SUVA, Freiwillige Akademische Gesellschaft, UBS Wealth Foundation, Talecris Biotherapeutics GmbH, Abbott Diagnostics, European Commission 018996 (GABRIEL), Wellcome Trust WT 084703MA. The study could not have been done without the help of the study participants, technical and administrative support and the medical teams and field workers at the local study sites. Local fieldworkers : Aarau: S Brun, G Giger, M Sperisen, M Stahel, Basel: C Bürli, C Dahler, N Oertli, I Harreh, F Karrer, G Novicic, N Wyttenbacher, Davos: A Saner, P Senn, R Winzeler, Geneva: F Bonfils, B Blicharz, C Landolt, J Rochat, Lugano: S Boccia, E Gehrig, MT Mandia, G Solari, B Viscardi, Montana: AP Bieri, C Darioly, M Maire, Payerne: F Ding, P Danieli A Vonnez, Wald: D Bodmer, E Hochstrasser, R Kunz, C Meier, J Rakic, U Schafroth, A Walder. Administrative staff: C Gabriel, R Gutknecht.

SHIP and **SHIP-TREND**. *The Study of Health in Pomerania*. SHIP is part of the Community Medicine Research net of the University of Greifswald, Germany, which is funded by the Federal Ministry of Education and Research (grants no. 01ZZ9603, 01ZZ0103, and 01ZZ0403), the Ministry of Cultural Affairs as well as the Social Ministry of the Federal State of Mecklenburg-West Pomerania, and the network

‘Greifswald Approach to Individualized Medicine (GANI_MED)’ funded by the Federal Ministry of Education and Research (grant 03IS2061A). Genome-wide data have been supported by the Federal Ministry of Education and Research (grant no. 03ZIK012) and a joint grant from Siemens Healthcare, Erlangen, Germany and the Federal State of Mecklenburg- West Pomerania. The University of Greifswald is a member of the ‘Center of Knowledge Interchange’ program of the Siemens AG and the Caché Campus program of the InterSystems GmbH. The SHIP authors are grateful to Mario Stanke for the opportunity to use his Server Cluster for the SNP imputation as well as to Holger Prokisch and Thomas Meitinger (Helmholtz Zentrum München) for the genotyping of the SHIP-TREND cohort.

TRAILS. *TRacking Adolescents' Individual Lives*. Trails is a collaborative project involving various departments of the University Medical Center and University of Groningen, the Erasmus University Medical Center Rotterdam, the University of Utrecht, the Radboud Medical Center Nijmegen, and the Parnassia Bavo group, all in the Netherlands. TRAILS has been financially supported by grants from the Netherlands Organization for Scientific Research NWO (Medical Research Council program grant GB-MW 940-38-011; ZonMW Brainpower grant 100-001-004; ZonMw Risk Behavior and Dependence grants 60-60600-98-018 and 60-60600-97-118; ZonMw Culture and Health grant 261-98-710; Social Sciences Council medium-sized investment grants GB-MaGW 480-01-006 and GB-MaGW 480-07-001; Social Sciences Council project grants GB-MaGW 457-03-018, GB-MaGW 452-04-314, and GB-MaGW 452-06-004; NWO large-sized investment grant 175.010.2003.005; NWO Longitudinal Survey and Panel Funding 481-08-013); the Sophia Foundation for Medical Research (projects 301 and 393), the Dutch Ministry of Justice (WODC), the European Science Foundation (EuroSTRESS project FP-006), and the participating universities. We are grateful to all adolescents, their parents and teachers who participated in this research and to everyone who worked on this project and made it possible. Statistical analyses were carried out on the Genetic Cluster Computer (http://www.geneticcluster.org), which is financially supported by the Netherlands Scientific Organization (NWO 480-05-003) along with a supplement from the Dutch Brain Foundation.

WGHS. *Women’s Genome Health Study*. The WGHS is supported by the National Heart, Lung, and Blood Institute (HL043851 and HL080467) and the National Cancer Institute (CA047988 and UM1CA182913), with collaborative scientific support and funding for genotyping provided by Amgen.

YFS. *Young Finns Study*. The YFS has been financially supported by the Academy of Finland: grants 134309 (Eye), 126925, 121584, 124282, 129378 (Salve), 117787 (Gendi), and 41071 (Skidi), the Social Insurance Institution of Finland, Kuopio, Tampere and Turku University Hospital Medical Funds (grant 9M048 and 9N035 for TeLeht), Juho Vainio Foundation, Paavo Nurmi Foundation, Finnish Foundation of Cardiovascular Research and Finnish Cultural Foundation, Tampere Tuberculosis Foundation and Emil Aaltonen Foundation (T.L). The technical assistance in the statistical analyses by Ville Aalto and Irina Lisinen is acknowledged.

References

1. Group, C.S. Vascular factors and risk of dementia: design of the Three-City Study and baseline characteristics of the study population. *Neuroepidemiology* **22**, 316-25 (2003).
2. Lambert, J.C. *et al.* Genome-wide association study identifies variants at CLU and CR1 associated with Alzheimer's disease. *Nat Genet* **41**, 1094-9 (2009).
3. Harris, T.B. *et al.* Age, Gene/Environment Susceptibility-Reykjavik Study: multidisciplinary applied phenomics. *Am J Epidemiol* **165**, 1076-87 (2007).
4. The Atherosclerosis Risk in Communities (ARIC) Study: design and objectives. The ARIC investigators. *Am J Epidemiol* **129**, 687-702 (1989).
5. Schmidt, R., Fazekas, F., Kapeller, P., Schmidt, H. & Hartung, H.P. MRI white matter hyperintensities: three-year follow-up of the Austrian Stroke Prevention Study. *Neurology* **53**, 132-9 (1999).
6. Schmidt, R. *et al.* Assessment of cerebrovascular risk profiles in healthy persons: definition of research goals and the Austrian Stroke Prevention Study (ASPS). *Neuroepidemiology* **13**, 308-13 (1994).
7. Attebo, K., Mitchell, P. & Smith, W. Visual acuity and the causes of visual loss in Australia. The Blue Mountains Eye Study. *Ophthalmology* **103**, 357-64 (1996).
8. Leeder, S.R. *et al.* Low hemoglobin, chronic kidney disease, and risk for coronary heart disease-related death: the Blue Mountains Eye Study. *J Am Soc Nephrol* **17**, 279-84 (2006).
9. Mitchell, P., Smith, W., Attebo, K. & Wang, J.J. Prevalence of age-related maculopathy in Australia. The Blue Mountains Eye Study. *Ophthalmology* **102**, 1450-60 (1995).
10. Firmann, M. *et al.* The CoLaus study: a population-based study to investigate the epidemiology and genetic determinants of cardiovascular risk factors and metabolic syndrome. *BMC Cardiovasc Disord* **8**, 6 (2008).
11. Nelis, M. *et al.* Genetic structure of Europeans: a view from the North-East. *PLoS One* **4**, e5472 (2009).
12. Higgins, M. *et al.* NHLBI Family Heart Study: objectives and design. *Am J Epidemiol* **143**, 1219-28 (1996).
13. Feinleib, M., Kannel, W.B., Garrison, R.J., McNamara, P.M. & Castelli, W.P. The Framingham Offspring Study. Design and preliminary data. *Prev Med* **4**, 518-25 (1975).
14. Kannel, W.B., Feinleib, M., McNamara, P.M., Garrison, R.J. & Castelli, W.P. An investigation of coronary heart disease in families. The Framingham offspring study. *Am J Epidemiol* **110**, 281-90 (1979).
15. Splansky, G.L. *et al.* The Third Generation Cohort of the National Heart, Lung, and Blood Institute's Framingham Heart Study: design, recruitment, and initial examination. *Am J Epidemiol* **165**, 1328-35 (2007).
16. Boger, C.A. *et al.* Association of eGFR-Related Loci Identified by GWAS with Incident CKD and ESRD. *PLoS Genet* **7**, e1002292 (2011).
17. Boger, C.A. *et al.* Effect of ACE and AT-2 inhibitors on mortality and progression to microalbuminuria in a nested case-control study of diabetic nephropathy in diabetes mellitus type 2: results from the GENDIAN study. *Int J Clin Pharmacol Ther* **44**, 364-74 (2006).
18. McEvoy, M. *et al.* Cohort profile: The Hunter Community Study. *Int J Epidemiol* **39**, 1452-63 (2010).
19. Hu, F.B. *et al.* Physical activity and television watching in relation to risk for type 2 diabetes mellitus in men. *Arch Intern Med* **161**, 1542-8 (2001).
20. Lin, J., Hu, F.B. & Curhan, G. Serum adiponectin and renal dysfunction in men with type 2 diabetes. *Diabetes Care* **30**, 239-44 (2007).

21. Manson, J.E. *et al.* Physical activity and incidence of non-insulin-dependent diabetes mellitus in women. *Lancet* **338**, 774-8 (1991).
22. Qi, L. *et al.* Genetic variants at 2q24 are associated with susceptibility to type 2 diabetes. *Hum Mol Genet* **19**, 2706-15 (2010).
23. Rimm, E.B. *et al.* Prospective study of alcohol consumption and risk of coronary disease in men. *Lancet* **338**, 464-8 (1991).
24. Tepper, B.J. *et al.* Variation in the bitter-taste receptor gene TAS2R38, and adiposity in a genetically isolated population in Southern Italy. *Obesity (Silver Spring)* **16**, 2289-95 (2008).
25. Sala, C. *et al.* Variation of hemoglobin levels in normal Italian populations from genetic isolates. *Haematologica* **93**, 1372-5 (2008).
26. D'Adamo, P. *et al.* Metabonomics and population studies: age-related amino acids excretion and inferring networks through the study of urine samples in two Italian isolated populations. *Amino Acids* **38**, 65-73 (2010).
27. Sorice, R. *et al.* Association of a variant in the CHRNA5-A3-B4 gene cluster region to heavy smoking in the Italian population. *Eur J Hum Genet* **19**, 593-6 (2011).
28. Siervo, M. *et al.* Angiogenesis and biomarkers of cardiovascular risk in adults with metabolic syndrome. *J Intern Med* **268**, 338-47 (2010).
29. Siervo, M. *et al.* Body mass index is directly associated with biomarkers of angiogenesis and inflammation in children and adolescents. *Nutrition* **28**, 262-6 (2012).
30. Ruggiero, D. *et al.* Genetics of VEGF serum variation in human isolated populations of cilento: importance of VEGF polymorphisms. *PLoS One* **6**, e16982 (2011).
31. Colonna, V. *et al.* Comparing population structure as inferred from genealogical versus genetic information. *Eur J Hum Genet* **17**, 1635-41 (2009).
32. Colonna, V. *et al.* Campora: a young genetic isolate in South Italy. *Hum Hered* **64**, 123-35 (2007).
33. Ciullo, M. *et al.* Identification and replication of a novel obesity locus on chromosome 1q24 in isolated populations of Cilento. *Diabetes* **57**, 783-90 (2008).
34. Ciullo, M. *et al.* New susceptibility locus for hypertension on chromosome 8q by efficient pedigree-breaking in an Italian isolate. *Hum Mol Genet* **15**, 1735-43 (2006).
35. Traglia, M. *et al.* Heritability and demographic analyses in the large isolated population of Val Borbera suggest advantages in mapping complex traits genes. *PLoS One* **4**, e7554 (2009).
36. Tayo, B.O. *et al.* Genetic background of patients from a university medical center in Manhattan: implications for personalized medicine. *PLoS One* **6**, e19166 (2011).
37. Baumeister, S.E. *et al.* Effect of chronic kidney disease and comorbid conditions on health care costs: A 10-year observational study in a general population. *Am J Nephrol* **31**, 222-9 (2010).
38. Wichmann, H.E., Gieger, C., Illig, T. & Group, M.K.S. KORA-gen--resource for population genetics, controls and a broad spectrum of disease phenotypes. *Gesundheitswesen* **67 Suppl 1**, S26-30 (2005).
39. Stolk, R.P. *et al.* Universal risk factors for multifactorial diseases: LifeLines: a three-generation population-based study. *Eur J Epidemiol* **23**, 67-74 (2008).
40. Scholtens, S. *et al.* Cohort Profile: LifeLines, a three-generation cohort study and biobank. *Int J Epidemiol* **44**, 1172-80 (2015).
41. Klijs, B. *et al.* Representativeness of the LifeLines Cohort Study. *PLoS One* **10**, e0137203 (2015).
42. Bild, D.E. *et al.* Multi-Ethnic Study of Atherosclerosis: objectives and design. *Am J Epidemiol* **156**, 871-81 (2002).
43. Pattaro, C. *et al.* The genetic study of three population microisolates in South Tyrol (MICROS): study design and epidemiological perspectives. *BMC Med Genet* **8**, 29 (2007).
44. Marroni, F., Grazio, D., Pattaro, C., Devoto, M. & Pramstaller, P. Estimates of genetic and environmental contribution to 43 quantitative traits support sharing of a homogeneous environment in an isolated population from South Tyrol, Italy. *Hum Hered* **65**, 175-82 (2008).

45. Lin, J., Fung, T.T., Hu, F.B. & Curhan, G.C. Association of dietary patterns with albuminuria and kidney function decline in older white women: a subgroup analysis from the Nurses' Health Study. *Am J Kidney Dis* **57**, 245-54 (2011).
46. Lin, J. & Curhan, G.C. Associations of sugar and artificially sweetened soda with albuminuria and kidney function decline in women. *Clin J Am Soc Nephrol* **6**, 160-6 (2011).
47. Colditz, G.A. & Hankinson, S.E. The Nurses' Health Study: lifestyle and health among women. *Nat Rev Cancer* **5**, 388-96 (2005).
48. Johansson, A., Vavruch-Nilsson, V., Edin-Liljegren, A., Sjolander, P. & Gyllensten, U. Linkage disequilibrium between microsatellite markers in the Swedish Sami relative to a worldwide selection of populations. *Hum Genet* **116**, 105-13 (2005).
49. Johansson, A., Vavruch-Nilsson, V., Cox, D.R., Frazer, K.A. & Gyllensten, U. Evaluation of the SNP tagging approach in an independent population sample--array-based SNP discovery in Sami. *Hum Genet* **122**, 141-50 (2007).
50. Hofman, A., Grobbee, D.E., de Jong, P.T. & van den Ouweland, F.A. Determinants of disease and disability in the elderly: the Rotterdam Elderly Study. *Eur J Epidemiol* **7**, 403-22 (1991).
51. Hofman, A. *et al.* The Rotterdam Study: objectives and design update. *Eur J Epidemiol* **22**, 819-29 (2007).
52. Hofman, A. *et al.* The Rotterdam Study: 2010 objectives and design update. *Eur J Epidemiol* **24**, 553-72 (2009).
53. Hofman, A. *et al.* The Rotterdam Study: 2016 objectives and design update. *Eur J Epidemiol* **30**, 661-708 (2015).
54. Volzke, H. *et al.* Cohort profile: the study of health in Pomerania. *Int J Epidemiol* **40**, 294-307 (2011).
55. John, U. *et al.* Study of Health In Pomerania (SHIP): a health examination survey in an east German region: objectives and design. *Soz Praventivmed* **46**, 186-94 (2001).
56. Ridker, P.M. *et al.* Rationale, design, and methodology of the Women's Genome Health Study: a genome-wide association study of more than 25,000 initially healthy american women. *Clin Chem* **54**, 249-55 (2008).
57. Patterson, N., Price, A.L. & Reich, D. Population structure and eigenanalysis. *PLoS Genet* **2**, e190 (2006).
58. Hutchison, N., Hendry, B.M. & Sharpe, C.C. Rho isoforms have distinct and specific functions in the process of epithelial to mesenchymal transition in renal proximal tubular cells. *Cell Signal* **21**, 1522-31 (2009).
59. Koshikawa, S., Nishikimi, T., Inaba, C., Akimoto, K. & Matsuoka, H. Fasudil, a Rho-kinase inhibitor, reverses L-NAME exacerbated severe nephrosclerosis in spontaneously hypertensive rats. *J Hypertens* **26**, 1837-48 (2008).
60. Schroeder, C. *et al.* PIK3R1 mutations in SHORT syndrome. *Clin Genet* **86**, 292-4 (2014).
61. Chasman, D.I. *et al.* Integration of genome-wide association studies with biological knowledge identifies six novel genes related to kidney function. *Hum Mol Genet* **21**, 5329-43 (2012).
62. Zheng, Z. *et al.* A Mendelian locus on chromosome 16 determines susceptibility to doxorubicin nephropathy in the mouse. *Proc Natl Acad Sci U S A* **102**, 2502-7 (2005).
63. Gascard, P. *et al.* Putative tumor suppressor protein 4.1B is differentially expressed in kidney and brain via alternative promoters and 5' alternative splicing. *Biochim Biophys Acta* **1680**, 71-82 (2004).
64. Kottgen, A. *et al.* Multiple loci associated with indices of renal function and chronic kidney disease. *Nat Genet* **41**, 712-7 (2009).
65. Pattaro, C. *et al.* Genetic associations at 53 loci highlight cell types and biological pathways relevant for kidney function. *Nat Commun* **7**, 10023 (2016).

66. Pattaro, C. *et al.* Genome-wide association and functional follow-up reveals new loci for kidney function. *PLoS Genet* **8**, e1002584 (2012).
67. Kottgen, A. *et al.* New loci associated with kidney function and chronic kidney disease. *Nat Genet* **42**, 376-84 (2010).
68. Willer, C.J., Li, Y. & Abecasis, G.R. METAL: fast and efficient meta-analysis of genomewide association scans. *Bioinformatics* **26**, 2190-1 (2010).
69. Olden, M. *et al.* Common variants in UMOD associate with urinary uromodulin levels: a meta-analysis. *J Am Soc Nephrol* **25**, 1869-82 (2014).
70. Shin, S.Y. *et al.* An atlas of genetic influences on human blood metabolites. *Nat Genet* **46**, 543-50 (2014).
71. Reznichenko, A. *et al.* SLC22A2 is associated with tubular creatinine secretion and bias of estimated GFR in renal transplantation. *Physiol Genomics* **45**, 201-9 (2013).
72. Yu, B. *et al.* Genetic determinants influencing human serum metabolome among African Americans. *PLoS Genet* **10**, e1004212 (2014).
73. Liu, C.T. *et al.* Genetic association for renal traits among participants of African ancestry reveals new loci for renal function. *PLoS Genet* **7**, e1002264 (2011).
74. Franceschini, N. *et al.* Generalization of associations of kidney-related genetic loci to American Indians. *Clin J Am Soc Nephrol* **9**, 150-8 (2014).
75. Meyer, T.E. *et al.* Genome-wide association studies of serum magnesium, potassium, and sodium concentrations identify six Loci influencing serum magnesium levels. *PLoS Genet* **6**(2010).
76. Cho, G.S., Choi, S.C., Park, E.C. & Han, J.K. Role of Tbx2 in defining the territory of the pronephric nephron. *Development* **138**, 465-74 (2011).
77. Yeo, N.C. *et al.* Shroom3 contributes to the maintenance of the glomerular filtration barrier integrity. *Genome Res* **25**, 57-65 (2015).
78. Khalili, H. *et al.* Developmental Origins for Kidney Disease Due to Shroom3 Deficiency. *J Am Soc Nephrol* (2016).
